# AL398, A Novel NSUN6 Inhibitor, Attenuates Epithelial‐Mesenchymal Transition and Metastasis in Endometrial Cancer by Abrogating m5C‐Dependent Stabilization of Snail mRNA

**DOI:** 10.1002/advs.77359

**Published:** 2026-08-28

**Authors:** Xiangzhuan Zhao, Linlin Yang, Huanxiang Chen, Chenfeng Guo, Hongshen Lu, Ying Zhu, Xuanzhi Wang, Yang Li, Ruike Zhang, Zichong Huang, Yifang Chen, Keyu Jin, Zeduo Xu, Lingxiu Meng, Longfeng Zhang, Yuanyuan Han, Yicong Yan, Qiaozhen Kang, Junhu Wan, Hongyang Liu

**Affiliations:** ^1^ Department of Obstetrics and Gynecology The Third Affiliated Hospital of Zhengzhou University Zhengzhou Henan China; ^2^ Department of Clinical Laboratory The First Affiliated Hospital of Zhengzhou University Zhengzhou Henan China; ^3^ School of Life Science Zhengzhou University Zhengzhou Henan China; ^4^ Department of Pharmacology School of Basic Medical Sciences Zhengzhou University Zhengzhou China; ^5^ School of Pharmacy Zhengzhou University Zhengzhou Henan China; ^6^ Department of Pathology The Third Provincial Hospital of Henan Zhengzhou Henan China

**Keywords:** EMT, endometrial cancer, m5C methylation, NSUN6, Snail

## Abstract

Endometrial cancer (EC) constitutes a leading gynecologic malignancy for which advanced or metastatic disease presents limited therapeutic options. While RNA modifications are acknowledged as key regulatory elements in cancer, the role of 5‐methylcytosine (m^5^C) and its modifying enzymes in EC is still largely unstudied. Here, it demonstrates that the m^5^C methyltransferase NSUN6 is significantly upregulated in EC tissues, and its expression correlates with higher tumor grade and unfavorable prognosis. Functional studies employing gain‐ and loss‐of‐function models revealed that NSUN6 specifically enhances EC cell migration and invasion both in vitro and in vivo, without altering proliferation or apoptosis. Mechanistically, NSUN6 drives EMT by catalyzing m^5^C modification on Snail mRNA, which in turn stabilizes the transcript of this key EMT transcription factor. AL398 was identified through structure‐based virtual screening as a novel small‐molecule inhibitor that binds potently to the NSUN6 active site and suppresses its methyltransferase activity. Treatment with AL398 effectively inhibited the NSUN6/m^5^C/Snail axis, reversed the EMT phenotype, and attenuated EC cell migration, invasion, and metastasis in preclinical models. Collectively, it both validates the NSUN6/m^5^C/Snail axis as a critical EC vulnerability and provides a proof‐of‐concept inhibitor, AL398, laying the groundwork for the development of first‐in‐class RNA methylation‐targeted therapies against metastatic EC.

## Graphical abstract

Proposed molecular mechanism: NSUN6 catalyzes m5C methylation on Snail mRNA to enhance its stability, leading to Snail upregulation and EMT promotion. The first‐in‐class inhibitor AL398 suppresses endometrial cancer metastasis by targeting the NSUN6‐m5C‐Snail. axis.

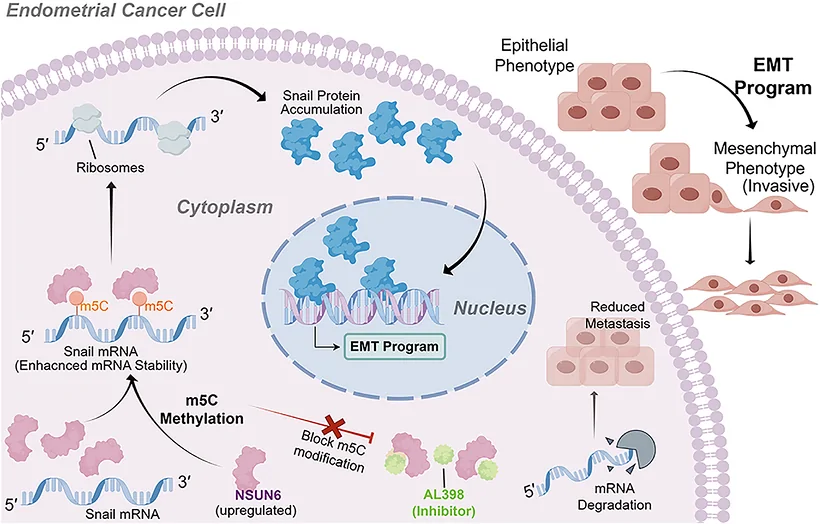

## 1 Introduction

Endometrial cancer is a cancerous growth that starts in the lining of the uterus and is the most prevalent gynecological cancer globally [1, 2]. Endometrial cancer (EC) ranks as the fourth most prevalent cancer among women, according to the 2025 Global Cancer Statistics, and accounts for 5% of all cancer cases [3]. Although most endometrial cancers are diagnosed at an early stage and exhibit excellent 5‐year survival rates‐exceeding 90% for stage I disease [4]. However, the prevailing notion that endometrial cancer is a benign tumor is profoundly misguided. Particularly, a subset of endometrial cancers, typified by uterine serous carcinoma, constitutes a distinct histological subtype. Including serous carcinoma, clear cell carcinoma, carcinosarcoma, mesothelioma, and leiomyosarcoma, these tumors display extremely aggressive biological behavior and have much worse clinical outcomes than typical low‐grade (G1‐G2) endometrial tumors [5]. Thus, the emergence of aggressive features marks a critical shift toward a refractory and lethal disease phenotype. Currently, the standard treatment for EC involves adjuvant therapy, including chemotherapy and/or radiotherapy, guided by the tumor's grade and stage after surgical intervention [6]. Yet, for patients with primary metastatic or recurrent endometrial carcinoma and a poor outlook, treatment choices remain limited. The current standard treatment is largely reliant on platinum‐based chemotherapy. Recently, the FDA approved the combination of Pembrolizumab and Lenvatinib for specific advanced endometrial cancer categories [7]. In light of the poor prognosis, restricted treatment options, and suboptimal outcomes for patients with primary metastatic or recurrent endometrial carcinoma, there is a critical need to further investigate the mechanisms underlying EC development and progression.

Targeting enzymes that modify RNA has emerged as a promising precision therapeutic strategy due to the growing understanding of RNA modifications. For example, inhibitors of m^6^A‐modifying enzymes such as FTO [8, 9, 10] and METTL3 [11, 12, 13] have shown considerable anti‐tumor potential. Another frequent post‐transcriptional RNA methylation is the m5C modification, which involves adding a methyl group to the fifth carbon of cytosine residues. However, inhibitors targeting m5C‐modifying enzymes remain scarce. The development of such novel targeted agents heavily relies on structure‐based approaches, including virtual screening and molecular docking against the three‐dimensional architecture of the enzyme of interest. NOP2/Sun RNA methyltransferase family member 6 (NSUN6) serves as the principal m5C methyltransferase responsible for m5C deposition on mammalian mRNA [14]. NSUN6‐mediated m5C modification promotes malignant progression across various cancers by regulating distinct downstream targets. NSUN6 enhances radioresistance in cervical cancer by elevating NDRG1 mRNA levels [15] In gastric cancer, it similarly enhances tumor development by stabilizing CEBPZ mRNA, relying on m5C [16]. In colon adenocarcinoma, NSUN6‐mediated modification of METTL3 further activates downstream oncogenic signaling pathways [17]. These findings illustrate how NSUN6 exerts tumor‐promoting functions through context‐specific molecular targets. However, the function of NSUN6 in EC progression and its underlying molecular mechanisms remain unclear, and there is a notable scarcity of reports on NSUN6 inhibitors.

The metastasis of tumors is a complex process involving multiple stages, governed by the coordinated actions of numerous molecular pathways. The cascade, involving the detachment of cells from the primary tumor, local invasion, circulation entry, and subsequent exit and growth at distant sites, is tightly controlled by an intricate molecular system. This process is largely influenced by the EMT, which causes tumor cells to lose epithelial traits and acquire a mesenchymal phenotype, enhancing their ability to migrate and invade. In this process, the transcription factor Snail serves as a key hub, whose expression and activity are precisely controlled at multiple levels. Snail is activated directly by the TGF‐β/Smad signaling pathway [18] and modulated by inflammatory mediators such as NF‐κB via specific promoter elements [19]. Post‐transcriptionally, m^6^A methylation, mediated by METTL3, regulates Snail mRNA stability, translation efficiency, and nucleocytoplasmic transport [20]. Phosphorylation and ubiquitination fine‐tune Snail protein stability, subcellular localization, and transcriptional activity; for example, GSK‐3β‐mediated phosphorylation promotes its proteasomal degradation [21]. However, despite the increasingly prominent role of RNA modification in tumor regulation, there is still a gap in systematic research concerning the role of m5C modification in the post‐transcriptional regulation of Snail and its mechanism in tumor metastasis.

In this study, we reveal a pro‐metastatic role for NSUN6 in endometrial cancer, mediated through m5C‐dependent stabilization of Snail mRNA and consequent EMT activation. Notably, we identify and validate AL398 as an inhibitor targeting NSUN6's methyltransferase activity, which potently reverses its oncogenic function. These results clarify an important RNA modification pathway in EC metastasis and suggest that NSUN6 could serve both as a prognostic biomarker and a therapeutic target in clinical settings.

## 2 Results

### 2.1 NSUN6 is Upregulated in Endometrial Cancer Tissues and Predicts Poor Prognosis

NSUN6 has been reported to be highly expressed in multiple tumor types [15, 16], yet its expression patterns and functional mechanisms in EC remain unclear. To systematically investigate the role of NSUN6 in EC development and progression, we performed an integrated bioinformatics analysis using several authoritative databases. First, pan‐cancer expression profiling based on the Clinical Proteome Tumor Analysis Consortium (CPTAC) data revealed that NSUN6 is significantly overexpressed in various cancers, including endometrial cancer (Figure 1A), providing a rationale for further focus on EC. Subsequent differential expression analysis within the CPTAC dataset demonstrated that NSUN6 levels were markedly elevated in EC tissues compared with normal endometrial samples (Figure 1B). Given the clinical relevance of patient outcomes, we also evaluated the prognostic significance of NSUN6 using TCGA data, which indicated that high NSUN6 expression is associated with poorer survival in EC patients (Figure 1C). To validate these findings experimentally, we conducted immunohistochemical (IHC) staining on 38 EC tissues and 12 normal endometrial samples. The results confirmed that NSUN6 expression was significantly upregulated in cancerous tissues compared to normal controls (Figure 1D,G). Furthermore, integrative analysis of the clinical data revealed that NSUN6 expression was positively correlated with higher tumor grade (Figure 1E) and associated with poorer overall survival (Figure 1F). These results suggest that NSUN6 may contribute not only to tumor initiation but also to malignant progression. Western blot analysis of five paired clinical specimens (tumor vs. adjacent non‐tumor tissue) further demonstrated elevated NSUN6 protein levels in EC tissues (Figure 1H), supporting its potential oncogenic role. Moreover, comprehensive analysis of Western blot and qRT‐PCR data across cell lines revealed that NSUN6 expression is consistently higher in EC cell lines compared to normal endometrial stromal cells (ESCs) (Figure 1I,J). Among these, Ishikawa and HEC‐1B cells exhibited the most robust and consistent NSUN6 upregulation at both protein and mRNA levels. Consequently, these two cell lines were selected as representative models for subsequent loss‐of‐function studies to ensure the reliability and clinical relevance of our findings. Collectively, these results indicate that NSUN6 is upregulated in endometrial cancer and that its elevated expression is potentially linked to unfavorable prognosis.

**FIGURE 1:**
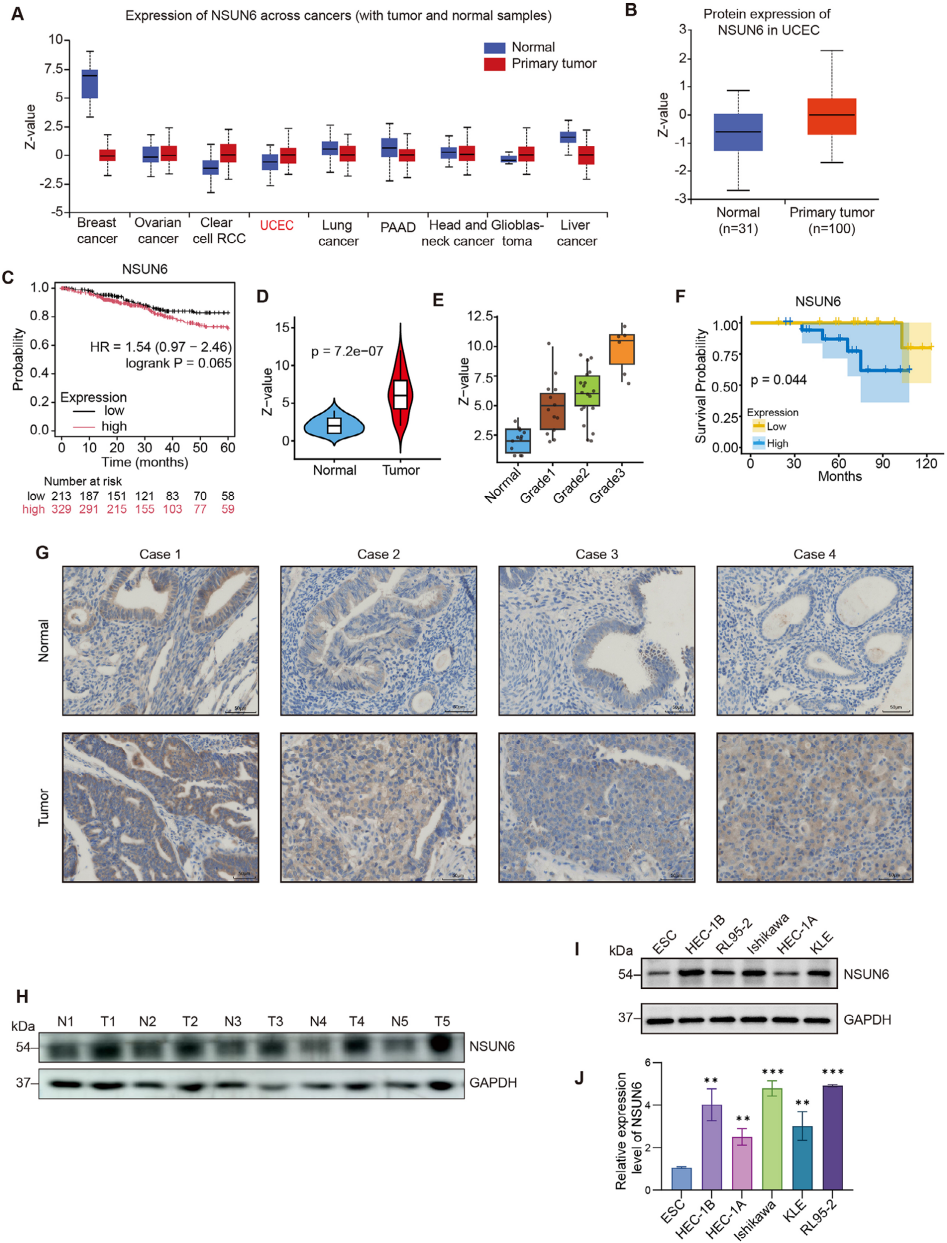
NSUN6 is upregulated in endometrial cancer tissues and predicts poor prognosis. (A) CPTAC database was used for pan‐cancer analysis of NSUN6. (B) CPTAC data analysis was performed to compare the expression level of NSUN6 between endometrial cancer tissues and normal tissues. (C) Kaplan‐Meier survival analysis was performed based on the TCGA dataset. (D) Comparison of NSUN6 expression between carcinoma and adjacent normal tissues. (E) The association analysis between tumor grade and NSUN6 expression level. (F) Association between NSUN6 expression and patient survival probability (*n* = 38 EC tissues). (G) Representative images show NSUN6 protein expression by IHC between carcinoma and adjacent normal tissues. (H) The protein expression levels of NSUN6 in carcinoma and adjacent normal tissues were compared by Western blotting. (I) Protein expression levels of NSUN6 in different cell lines. (J) The expression levels of NSUN6 in normal and endometrial cancer cell lines were detected by RT‐qPCR. All quantitative data are presented as mean ± SD from three independent experiments. ^*^ *p* < 0.05, ^**^ *p* < 0.01, ^***^ *p* < 0.001.

### 2.2 NSUN6 Promotes Migration and Invasion of Endometrial Cancer Cells Without Affecting Proliferation, Cell Cycle Progression, or Apoptosis

To assess the biological significance of NSUN6 in endometrial carcinoma progression, we first established NSUN6‐overexpressing and NSUN6‐knockdown models in Ishikawa and HEC‐1B cells, respectively. The efficiency of overexpression and knockdown was validated by RT‐qPCR and Western blot analysis (Figure 2A–D). We next performed a series of in vitro functional assays to evaluate the influence of NSUN6 on malignant behaviors. Wound healing assays revealed that NSUN6 overexpression markedly enhanced the migratory ability of endometrial carcinoma cells, as reflected by accelerated wound closure (Figure 2E,F). Similarly, Transwell migration assays and Matrigel‐based invasion assays demonstrated that NSUN6 overexpression significantly potentiated both migratory and invasive capacities. Conversely, NSUN6 knockdown attenuated these phenotypes (Figure 2G,H). We further investigated whether NSUN6 influences cell proliferation using CCK‐8, Edu assays, and colony formation. Results from the CCK‐8 assay indicated that neither NSUN6 overexpression nor knockdown exerted a statistically significant effect on proliferation rates in Ishikawa or HEC‐1B cells (Figure 3A,B). The Edu assay, which measures the proportion of proliferating cells based on Edu incorporation, further confirmed that NSUN6 expression does not significantly affect the proliferation rate of endometrial carcinoma cells (Figure 3C,D). Consistent with this, colony formation assays showed no correlation between NSUN6 expression levels and colony‐forming ability (Figure 3E). Subsequently, we examined cell cycle distribution and apoptosis. Flow cytometric analysis revealed no significant difference in the proportion of S‐phase cells upon modulation of NSUN6 expression (Figure 3F). Moreover, Annexin V/PI double‐staining combined with flow cytometry showed that neither NSUN6 overexpression nor knockdown significantly altered the apoptotic rate in Ishikawa cells (Figure 3G). Collectively, these results demonstrate that NSUN6 specifically promotes the migratory and invasive capabilities of endometrial carcinoma cells without exerting significant effects on proliferation, cell cycle progression, or apoptosis.

**FIGURE 2:**
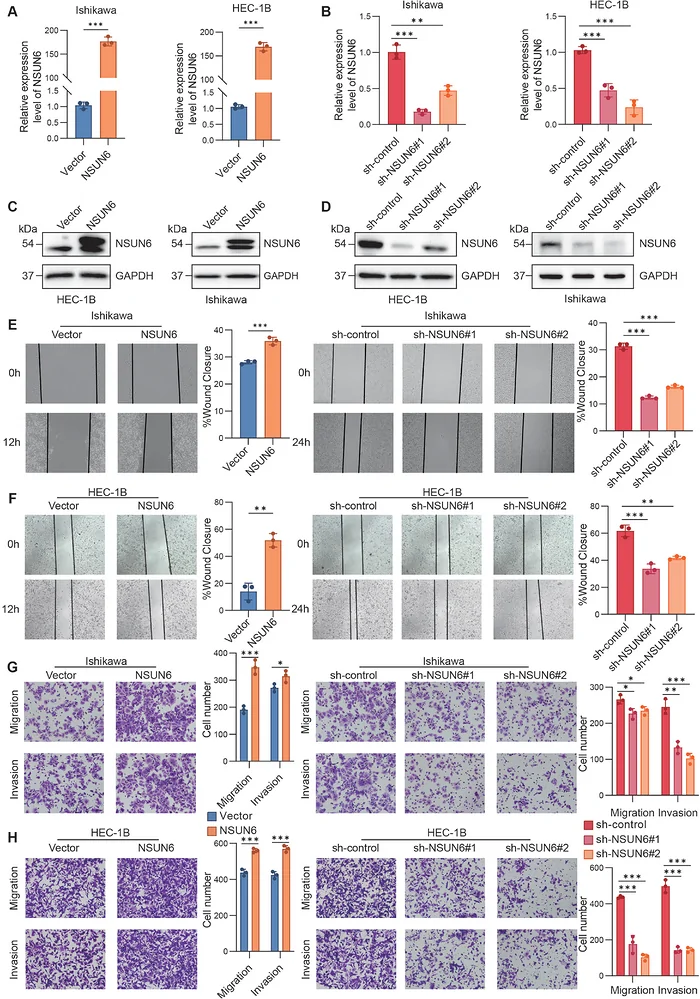
NSUN6 promotes migration and invasion of endometrial cancer cells. A‐D RT‐qPCR (A‐B) and Western blot (C‐D) were used to verify the overexpression and knockdown of NSUN6 in Ishikawa and HEC‐1B cells at the mRNA and protein levels, respectively. E‐F The migration ability of Ishikawa (E) and HEC‐1B (F) cells after overexpression and knockdown of NSUN6 was determined by wound healing assay. Representative images are shown on the left and statistical data analysis on the right. G‐H Transwell assay was used to detect the effect of NSUN6 overexpression and knockdown on the migration and invasion of Ishikawa. (G) and HEC‐1B (H) cells. Representative images are shown on the left and statistical data analysis on the right. All quantitative data are presented as mean ± SD from three independent experiments. ^*^ *p* < 0.05, ^**^ *p* < 0.01, ^***^ *p* < 0.001.

**FIGURE 3:**
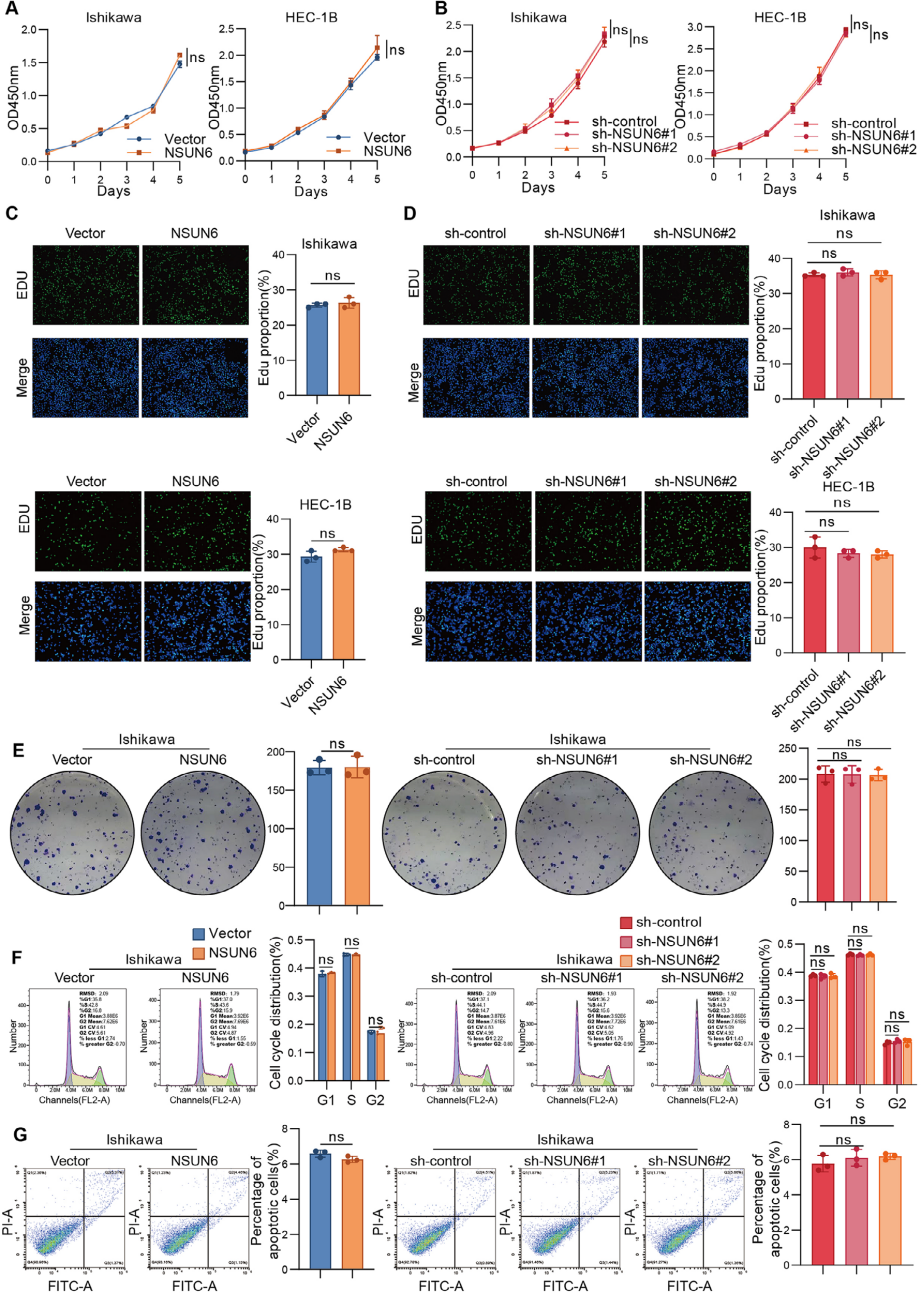
NSUN6 has no significant impact on the proliferation, cell cycle progression, or apoptosis of endometrial cancer cells. (A,B) The CCK‐8 assay was employed to assess the impact of NSUN6 overexpression (A) and knockdown (B) on cell proliferation in Ishikawa and HEC‐1B cell lines. C‐E EdU (C,D) and colony formation assays (E) were utilized to evaluate the effects of NSUN6 overexpression and knockdown on cell proliferation in these cell lines. Representative images are presented on the left, with corresponding statistical data analyses on the right. (F,G) Flow cytometry was conducted to examine the cell cycle (F) and apoptosis (G) in Ishikawa cells. All quantitative data are presented as mean ± SD from three independent experiments. ^*^ *p* < 0.05, ^**^ *p* < 0.01, ^***^ *p* < 0.001.

### 2.3 Single‐Cell Profiling Identifies Heterogeneous NSUN6 Expression and Implicates Snail Regulation in Endometrial Cancer

Single‐cell resolution facilitates the characterization of heterogeneity within tumor cell populations. To investigate whether NSUN6 expression varies among endometrial cancer cells at this resolution, we analyzed single‐cell sequencing data from 5 cases of endometrial cancer tissue from GEO dataset GSE173682. After quality control of the scRNA‐seq data‐including filtering, standardization, and normalization‐highly variable genes were identified. Principal component analysis was performed, and significant dimensions were selected based on cumulative variance explained. Cell clustering was carried out using the FindClusters function, and each cluster was manually annotated according to canonical marker genes. Six major cell types were identified: endothelial cells, epithelial cells, monocyte‐macrophages, fibroblasts, parietal cells, and T cells (Figure 4A).

**FIGURE 4:**
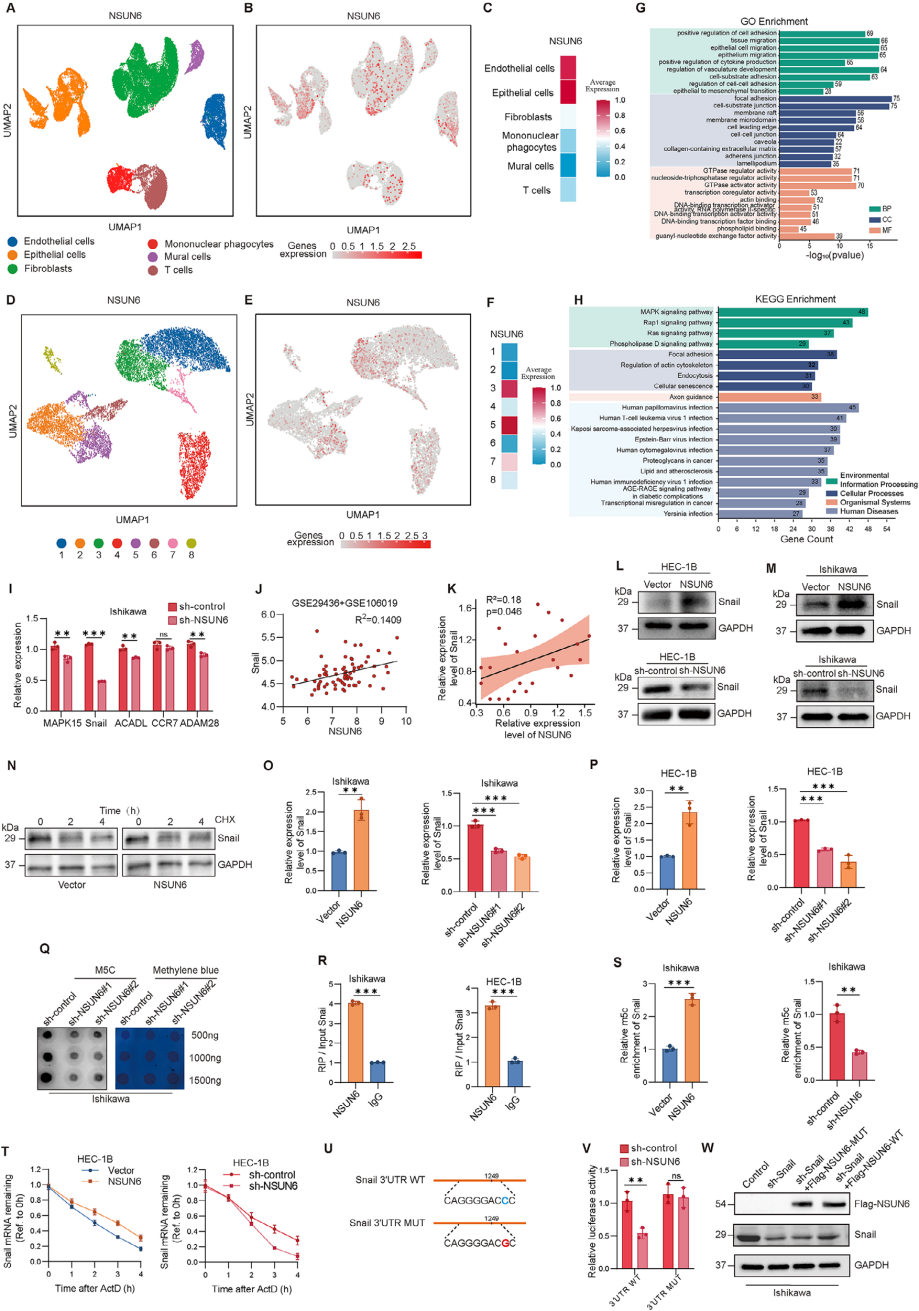
Single‐cell Profiling Identifies Heterogeneous NSUN6 Expression and Implicates Snail Regulation in Endometrial Cancer. (A) UMAP of major cell types (initial clustering). B Expression distribution of NSUN6 across major cell types (UMAP). (C) Heatmap of NSUN6 expression across major cell types. (D–F) Sub‐analysis of merged epithelial and endothelial cells with high NSUN6 expression. (D) UMAP of epithelial/endothelial subclusters (sub‐clustering). (E) Expression distribution of NSUN6 across epithelial/endothelial subclusters (UMAP). (F) Heatmap of NSUN6 expression across epithelial/endothelial subclusters. (G,H) Functional enrichment analysis of differentially expressed genes between NSUN6‐high and ‐low subclusters. Differential gene expression analysis between NSUN6‐high and ‐low subclusters (defined by secondary clustering) was performed, and all DEGs were subjected to pathway enrichment analysis. (G) Enriched GO biological processes for the DEGs. (H) Enriched KEGG pathways for the DEGs. (I) The mRNA levels of five differentially expressed candidate genes were measured by RT‐qPCR in control and NSUN6‐overexpressing Ishikawa cells. (J) Correlation between NSUN6 and Snail expression in public endometrial carcinoma datasets. (K) Positive correlation between NSUN6 and Snail expression in our clinical cohort (*n* = 23 EC tissues). (L) Western blot analysis of Snail protein levels in HEC‐1B cells after overexpression and knockdown of NSUN6. (M) Western blot analysis of Snail protein levels in Ishikawa cells after overexpression and knockdown of NSUN6. (N) Ishikawa cells in the control group and NSUN6 overexpression group were treated with 100 µg/mL CHX for the indicated times, and protein expression of Snail was analyzed by Western blot analysis. (O) RT‐qPCR analysis of Snail mRNA levels in Ishikawa cells after overexpression and knockdown of NSUN6. (P) RT‐qPCR analysis of Snail mRNA levels in HEC‐1B cells after overexpression and knockdown of NSUN6. (Q) Dot Blot analysis of global m5C methylation in total RNA after NSUN6 knockdown in Ishikawa cells. Methylene Blue staining served as the RNA loading control. (R) The results of RIP and RT‐qPCR in Ishikawa and HEC‐1B cells. IgG was used as a negative control to preclude nonspecific binding. (S) The results of methylated‐RIP for Ishikawa and HEC‐1B cells. The relative m5 C enrichment of Snail mRNA for each group was normalized to the Input. (T) The relative expression of Snail mRNA was detected after treating Ishikawa and HEC‐1B cells with 5 µg/mL actinomycin D for indicated times. (U) Schematic of Snail 3′UTR wild‐type and mutant constructs for binding site verification. (V) Dual‐luciferase reporter assay in cells transfected with Snail 3′UTR WT or MUT constructs, grouped by sh‐control or sh‐NSUN6 treatment. (W) Western blot analysis of Snail protein levels in Ishikawa cells across different experimental groups (Control, sh‐Snail, sh‐Snail + Flag‐NSUN6‐MUT, and sh‐Snail + Flag‐NSUN6‐WT). All quantitative data are presented as mean ± SD from three independent experiments. ^*^ *p* < 0.05, ^**^ *p* < 0.01, ^***^ *p* < 0.001.

Initial analysis revealed differential NSUN6 expressions across these cell types, with notably higher levels in epithelial and endothelial cells (Figure 4B,C). To further explore the role of NSUN6 in endometrial cancer, we subsetted epithelial and endothelial cells and repeated normalization, dimensionality reduction, and clustering. These cells segregated into eight distinct subpopulations (Figure 4D). Among them, NSUN6 expression was significantly elevated in subpopulations 3 and 5, while remaining low in subpopulations 1, 2, and 6 (Figure 4E,F). Focusing on subpopulations 5 (highest NSUN6 expression) and 1 (lowest expression), which exhibited the most pronounced difference, we performed differential gene expression analysis. GO and KEGG enrichment analyses demonstrated that differentially expressed genes were enriched in functional modules and signaling networks driving tumor invasion and metastasis, including epithelial‐mesenchymal transition, cell migration and adhesion, and actin cytoskeleton regulation (Figure 4G,H). To investigate downstream mechanisms by which NSUN6 influences tumor cell migration and invasion, we screened five metastasis‐related differentially expressed genes. RT‐qPCR validation following NSUN6 knockdown revealed the most significant downregulation in Snail mRNA expression (Figure 4I). Therefore, subsequent studies will focus on NSUN6‐mediated regulation of Snail.

### 2.4 NSUN6‐Mediated m5C Modification of Snail mRNA Maintains its Stability

Analysis of the GEO endometrial cancer transcriptome database confirmed that NSUN6 expression was positively correlated with Snail (Figure 4J). To investigate the regulatory relationship between NSUN6 and Snail in clinical settings, we extracted total RNA from endometrial cancer tissue specimens and performed RT‐qPCR analysis to quantify the expression levels of both genes. The results demonstrated a significant positive correlation between NSUN6 and Snail expression in these clinical samples (Figure 4K), prompting further investigation into the regulatory mechanism. Western blot analysis demonstrated that knockdown or overexpression of NSUN6 significantly decreased or increased Snail protein levels, respectively (Figure 4L,M). To exclude potential effects on protein degradation, cells were treated with cycloheximide (CHX). Neither NSUN6 knockdown nor overexpression substantially altered Snail protein stability (Figure 4N), suggesting regulation at the transcriptional or post‐transcriptional level.

Consistent with this, RT‐qPCR confirmed that Snail mRNA levels were significantly reduced upon NSUN6 knockdown (Figure 4O,P). Dot blot analysis indicated a global decrease in transcriptomic m5C levels following NSUN6 depletion, confirming its functional role as an m5C methyltransferase (Figure 4Q). Given the methyltransferase activity of NSUN6, we examined whether it directly modifies Snail mRNA. RNA immunoprecipitation (RIP) confirmed binding of NSUN6 protein to Snail mRNA (Figure 4R). Subsequent m5C methylated RNA immunoprecipitation (MeRIP‐qPCR) revealed that NSUN6 overexpression enhanced, while its knockdown reduced, m5C modification on Snail mRNA (Figure 4S), indicating specific catalytic activity toward this transcript. To assess the functional consequence of this modification, actinomycin D (Act D)‐based mRNA stability assays were performed. Overexpression of NSUN6 extended the half‐life of Snail mRNA, whereas knockdown accelerated its decay (Figure 4T). To specifically validate the target site, we constructed a luciferase reporter plasmid containing the wild‐type (WT) or mutated (MUT) Snail 3′UTR. The mutation site was selected based on the prediction from the RNAm5Cfinder database, where the m5C‐modified cytosine at position 1249 was substituted (Figure 4U). Dual‐luciferase reporter assays demonstrated that NSUN6 knockdown significantly reduced the luciferase activity of the Snail‐WT reporter but had no significant effect on the Snail‐MUT reporter (Figure 4V). These results indicate that NSUN6 directly targets the 1249 site of Snail mRNA for m5C modification.

Given that m5C modifications often recruit “reader” proteins to regulate RNA metabolism, we investigated potential mediators by performing RNA immunoprecipitation (RIP) assays for common m5C readers, specifically YBX1 and ALYREF. RIP assays confirmed that YBX1, but not ALYREF, specifically binds to Snail mRNA (Figure S1C). Actinomycin D assays further demonstrated that YBX1 overexpression increased Snail mRNA stability, while its knockdown had the opposite effect (Figure S1D). Importantly, rescue experiments showed that overexpression of YBX1 could reverse the decrease in Snail protein levels and the impairment of cell migration and invasion induced by NSUN6 knockdown (Figure S1E,F). Collectively, these data identify YBX1 as the functional reader protein that recognizes the m5C modification on Snail mRNA to enhance its stability and promote cell malignancy.

To further confirm that the catalytic activity of NSUN6 is essential for this regulation, we generated an NSUN6‐MUT mutant by substituting the conserved catalytic cysteine at position 373 with alanine. We performed rescue experiments by re‐introducing either wild‐type NSUN6 (Flag‐NSUN6‐WT) or the catalytic mutant (Flag‐NSUN6‐MUT) into Snail‐knockdown (sh‐Snail) Ishikawa cells. Western blot analysis revealed that while the re‐introduction of Flag‐NSUN6‐WT could effectively rescue Snail protein levels, the catalytic mutant Flag‐NSUN6‐MUT failed to restore Snail expression (Figure 4W). These results indicate that the m5C methyltransferase activity of NSUN6 is critical for its positive regulation of Snail in endometrial cancer cells. These findings define a novel NSUN6/m5C/YBX1/Snail axis, highlighting that NSUN6‐mediated m5C methylation of Snail mRNA serves as a crucial determinant for YBX1‐dependent mRNA stabilization and the subsequent malignant progression of endometrial cancer.

### 2.5 NSUN6 Drives Epithelial‐Mesenchymal Transition by Regulating Snail in EC Cells

Given the pronounced effects of NSUN6 on the migration and invasion of endometrial cancer cells, together with the established role of Snail as a key regulator of EMT, we hypothesized that NSUN6 may promote EMT in endometrial cancer. Initially, we observed that NSUN6‐overexpressing Ishikawa and HEC‐1B cells displayed enlarged cell size, reduced polarity, spindle‐like morphology, and weakened intercellular adhesion compared with control cells (Figure 5A). We then evaluated the expression of EMT‐related markers by Western blot. The results showed that NSUN6 overexpression downregulated E‐cadherin and upregulated Snail, ZEB1, N‐cadherin, and vimentin, whereas NSUN6 knockdown produced the opposite effects (Figure 5B,C). Immunofluorescence (IF) staining further corroborated the promotive role of NSUN6 in EMT (Figure 5D–G). To further elucidate the role of Snail in NSUN6‐induced EMT, we knocked down Snail in endometrial cancer cells (Ishikawa, HEC‐1B) overexpressing NSUN6. We found that the absence of Snail significantly weakened the regulation of EMT markers such as E‐cadherin, N‐cadherin, and Vimentin by NSUN6. Conversely, replenishing Snail in cells with knocked‐down NSUN6 partially reversed the suppression of EMT phenotype caused by the absence of NSUN6. These reciprocal experiments indicate that Snail is located downstream of NSUN6 and serves as a key effector molecule regulating the progression of EMT (Figure 6A,B). We subsequently performed functional assays to assess the phenotypic consequences. The wound healing and Transwell assay results showed that overexpression of NSUN6 significantly promoted the migration and invasion abilities of endometrial cancer cells (Ishikawa, HEC‐1B), while knockdown of Snail significantly attenuated this promoting effect. Further overexpression of Snail in NSUN6‐knockdown cells revealed that it could partially restore the decreased migration and invasion abilities caused by the absence of NSUN6 (Figure 6C–H). In summary, these findings demonstrate that NSUN6 promotes endometrial cancer progression by activating Snail‐dependent EMT, and this pro‐tumorigenic effect is abrogated by Snail deficiency.

**FIGURE 5:**
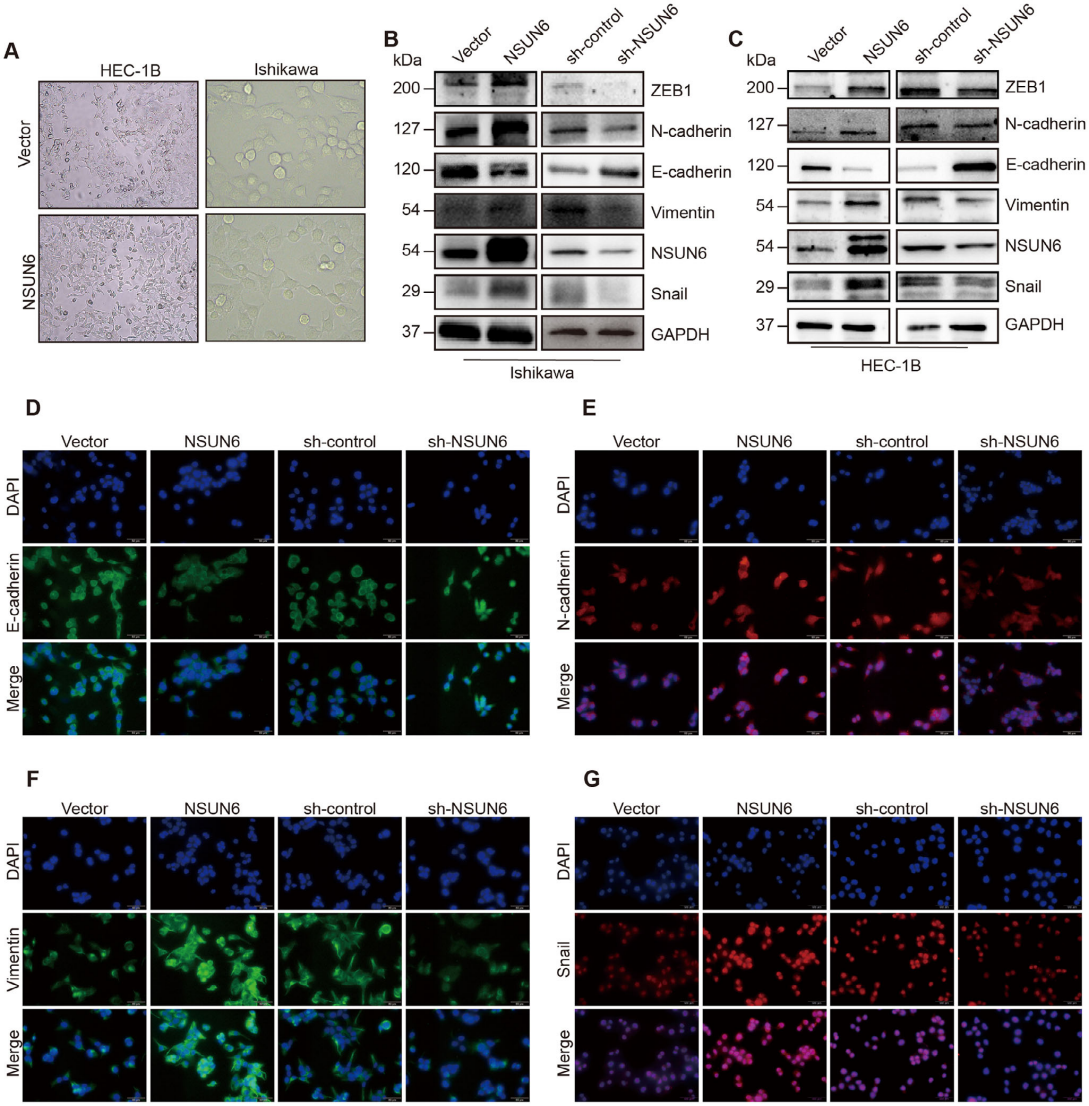
NSUN6 drives Epithelial‐mesenchymal transition by regulating Snail in EC cells. (A) The morphology of Ishikawa and HEC‐1B cells in the control and NSUN6‐overexpression groups was observed using a microscope. (B,C) The expression levels of the EMT proteins in Ishikawa (B) and HEC‐1B (C) cells from the control, NSUN6‐overexpression, and NSUN6‐knockdown groups were detected by western blot. (D–G) The effect of NSUN6 on the expression of different EMT markers in Ishikawa cells was detected by immunofluorescence.

**FIGURE 6:**
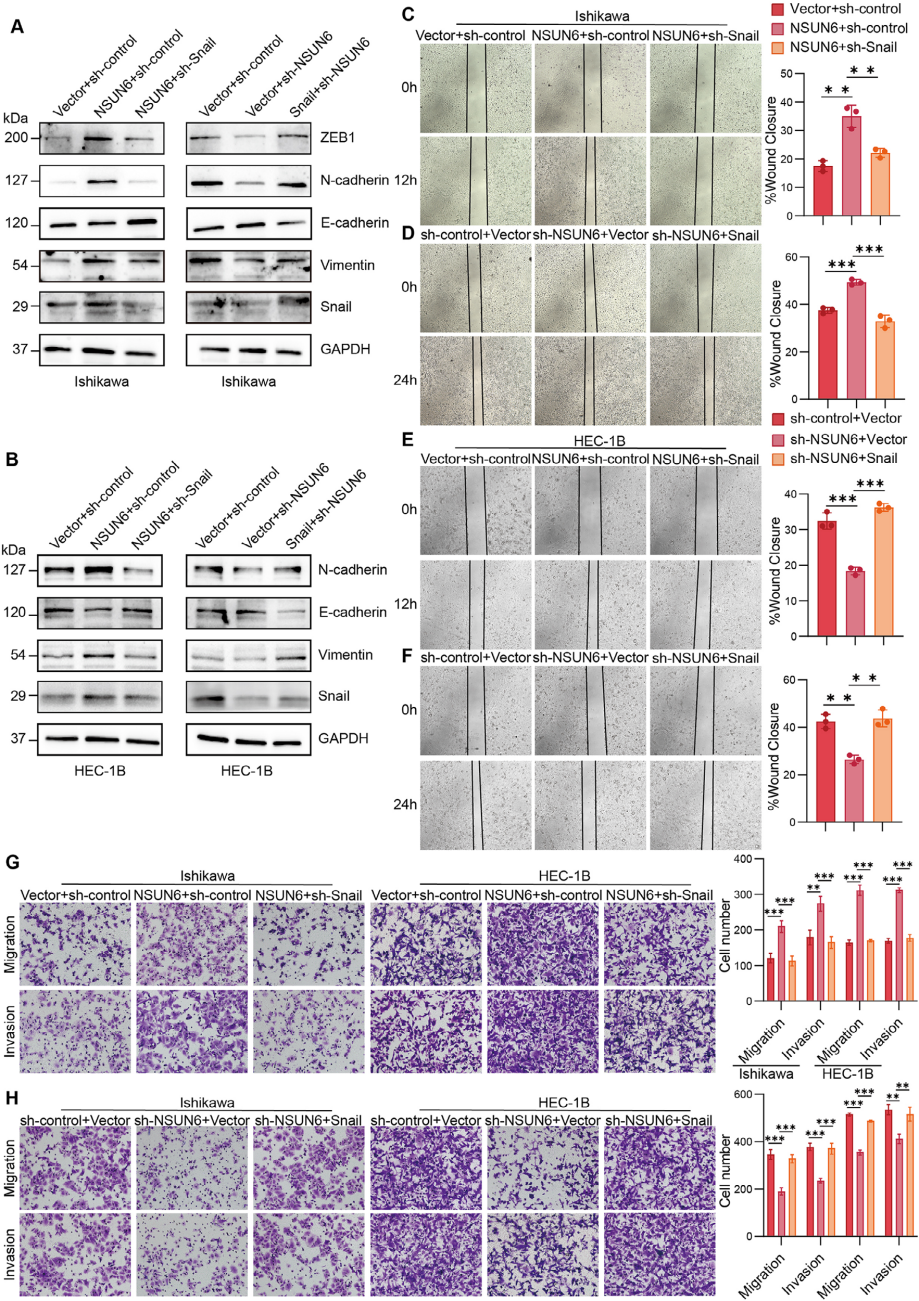
Knockdown of Snail rescues the pro‐metastatic phenotype induced by NSUN6 overexpression. A‐B Rescue validation in Ishikawa cells: (A) Snail knockdown reversed the regulation of EMT marker by NSUN6 overexpression, Snail replenishment partially restored the EMT phenotype upon NSUN6 knockdown; (B) The same set of experiments was repeated in HEC‐1B cells with consistent results. (C,D) In Ishikawa cells, NSUN6 overexpression promoted migration, which was reversed by Snail knockdown (C); NSUN6 knockdown inhibited migration, and this phenotype was partially rescued by Snail replenishment (D). (E,F) Consistent results were obtained in HEC‐1B cells. (G,H) Cell migration and invasion abilities were evaluated by Transwell assay in Ishikawa and HEC‐1B cells. All quantitative data are presented as mean ± SD from three independent experiments. ^*^ *p* < 0.05, ^**^ *p* < 0.01, ^***^ *p* < 0.001.

### 2.6 Structure‐Based Discovery and Characterization of a Novel NSUN6 Inhibitor, AL398

To identify potential NSUN6 inhibitors, this study employed a structure‐based drug design strategy. NSUN6 is an S‐adenosylmethionine (SAM)‐dependent methyltransferase. During catalysis, SAM is converted to S‐adenosylhomocysteine (SAH), which itself acts as a physiological inhibitor of this enzyme class. Sinefungin (SFG), a natural nucleoside analogue derived from *Streptomyces griseus*, structurally mimics SAH and can competitively bind to the SAM/SAH active pocket of NSUN6. The three‐dimensional structure of this complex has been resolved (PDB: 5WWR). Here, we targeted the active site of this structure for virtual screening to discover novel compounds capable of binding to this site and exhibiting inhibitory activity. Initial compounds were obtained from the SPECS commercial library containing 210331 molecules. Following preprocessing and optimization using the LigPrep tool, 294591 molecules were generated. Subsequently, the three‐dimensional protein structure was resolved based on 5WWR and completed using modeller. Then, the 290000 conformations obtained were scored through high‐throughput virtual screening (HTVS), and the first 10000 scored molecules were selected for standard precision (SP) screening for further refinement. Finally, 9856 conformations were obtained by removing repetitive molecules. Subsequently, Lipinsky's Five Rules were applied as filters, and 1500 compounds corresponding to the key features of 5WWR were selected. Cluster analysis was conducted based on structural features, and the highest‐ranked compound was selected from each cluster, generating a total of 119 candidate compounds (Figure 7A). Next, through visual observation and comparison of the binding patterns, based on the extension direction of the compound in the pocket, six molecules were ultimately obtained (Figure S2A–F). After obtaining these six preferred molecules, to further evaluate their binding strength and specificity with the target, we systematically analyzed the hydrogen bond network formed between each molecule and the NSUN6 protein structure. By comparing the number of hydrogen bonds, we selected the top two molecules (AL398 and AP770) with the largest number of hydrogen bonds formed with the active pocket as candidate compounds for further research.

**FIGURE 7:**
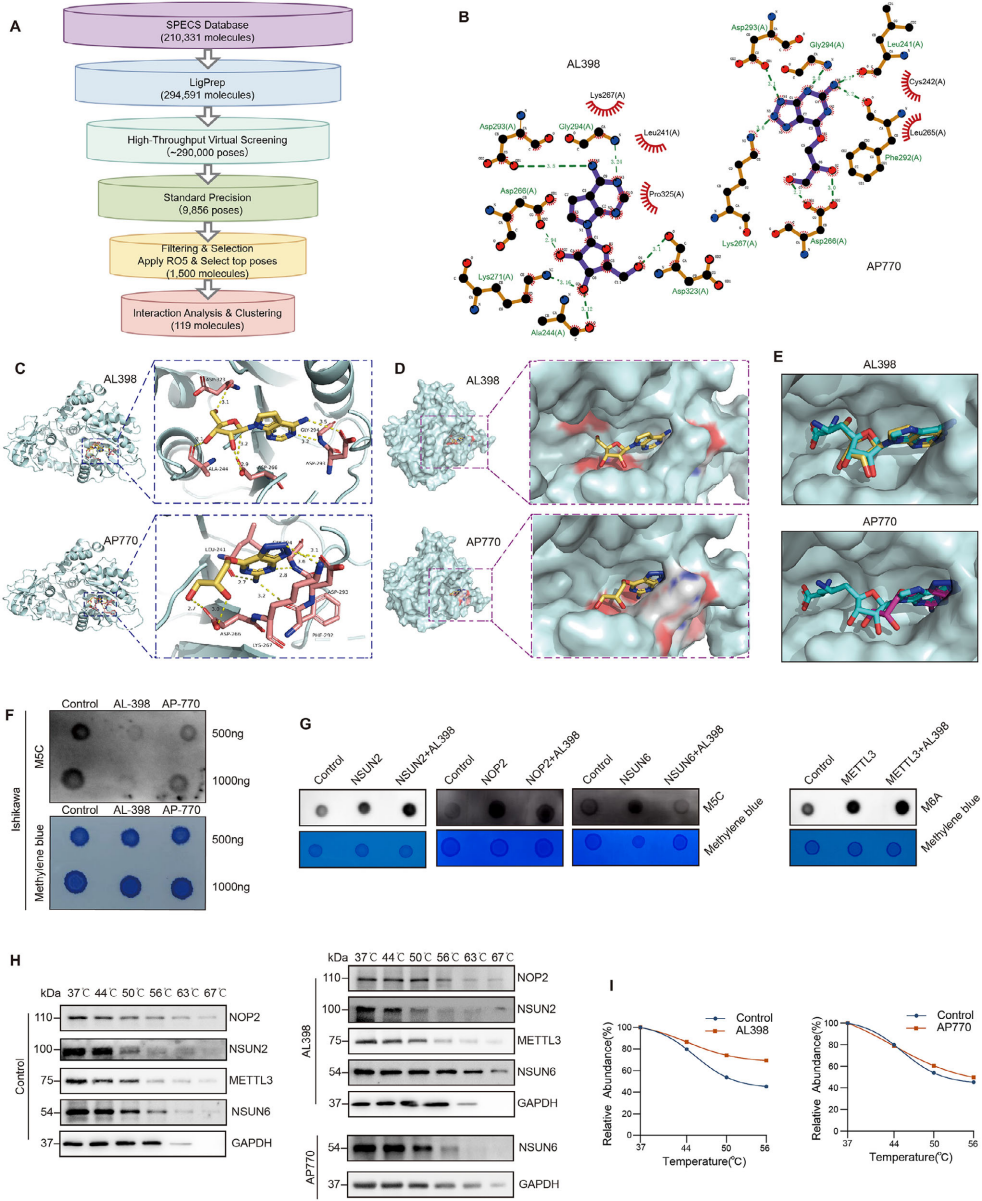
Structure‐based discovery and characterization of a novel NSUN6 inhibitor, AL398. (A) Virtual screening and docking workflow. (B) 2D diagram of molecular docking. (C) 3D diagram of molecular docking. (D) AL398 and AP770 are located in the active pocket of NSUN6. E AL398, AP770 and SFG exist in similar pockets. (F) m5C Investigation of m5C Expression in Ishikawa Cells Subsequent to Inhibitor Administration via the m5C Dot Hybridization Assay. (G) Dot blot assay to examine the effect of AL398 treatment on m5C or m6A levels. In addition to NSUN6 overexpression, other groups were performed by overexpressing the indicated methyltransferases in NSUN6‐knockdown cell lines (methylene blue staining served as the total RNA loading control). (H) Cellular thermal shift assay (CETSA) was performed to assess the effect of AL398 or AP770 treatment on the thermal stability of the target proteins. (I) Quantitative analysis of relative protein abundance from the cellular thermal shift assay (CETSA) showing the thermal stability curves upon drug treatment. All quantitative data are presented as mean ± SD from three independent experiments. ^*^ *p* < 0.05, ^**^ *p* < 0.01, ^***^ *p* < 0.001.

To identify more effective inhibitors, molecular docking studies were conducted on these two compounds using Autodock software. The NSUN6 protein structure (PDB: 5WWR) was retrieved from the RCSB Protein Data Bank. Docking analysis revealed that both AL398 and AP770 bind stably to the active pocket of NSUN6 and form hydrogen‐bond interactions with several key residues (Figure 7C,D). Specifically, AL398 interacts with Asp293, Asp266, Lys271, Ala244, Asp323, and Gly294, while AP770 forms hydrogen bonds with Asp239, Lys267, Asp266, Phe292, Leu241, and Gly294 (Figure 7B). The docking results indicated that the reference molecule SFG‐an endogenous inhibitor of NSUN6‐adopted a binding pose similar to that of AL398, with both occupying the same conserved active pocket. In contrast, the binding position of AP770 deviated considerably from the ideal conformation. AL398 was superior to AP770 in terms of both molecular conformation and interaction patterns (Figure 7E).

To verify the cellular activity of the candidate compounds, endometrial cancer (EC) cells were treated with each compound. Dot‐blot hybridization analysis of mRNA m5C levels revealed that AL398 reduced this modification more effectively than AP770 (Figure 7F). Based on these results and molecular docking analysis, AL398 was selected as the candidate inhibitor targeting NSUN6. Furthermore, cellular thermal shift assay (CETSA) demonstrated that AL398 treatment significantly enhanced the thermal stability of the NSUN6 protein in EC cells (Figure 7I), while showing negligible effects on the stability of other methyltransferases, including NOP2, NSUN2, and METTL3 (Figure 7H and Figure S2H). Notably, dot‐blot assays further confirmed that AL398 specifically inhibits m5C modification mediated by NSUN6, but not by NOP2 or NSUN2, nor does it affect m6A modification by METTL3 (Figure 7G). Mechanistically, MeRIP‐qPCR assay confirmed that AL398 treatment effectively reverses the NSUN6‐induced increase in both the m5C modification level (Figure S1B). Collectively, these multi‐faceted screenings and validations identify AL398 as a novel and highly specific NSUN6 inhibitor.

### 2.7 AL398 Attenuates Metastatic Potential of Endometrial Cancer Cells In Vitro

To elucidate the potential role of AL398 in endometrial carcinoma cells, Ishikawa and HEC‐1B cells were treated with AL398 at varying concentrations (0, 3, and 6 µm). We then employed wound healing and Transwell migration assays to evaluate the migratory capacity of these cells. The results demonstrated that AL398 significantly suppressed the migration of endometrial carcinoma cells in a dose‐dependent manner. Furthermore, cell invasion was assessed using Transwell matrix penetration assays. Compared with the respective control groups, AL398‐treated cells exhibited a marked reduction in invasive ability. Collectively, these findings suggest that AL398 negatively regulates the progression of endometrial carcinoma cells in vitro (Figure 8A–D).

**FIGURE 8:**
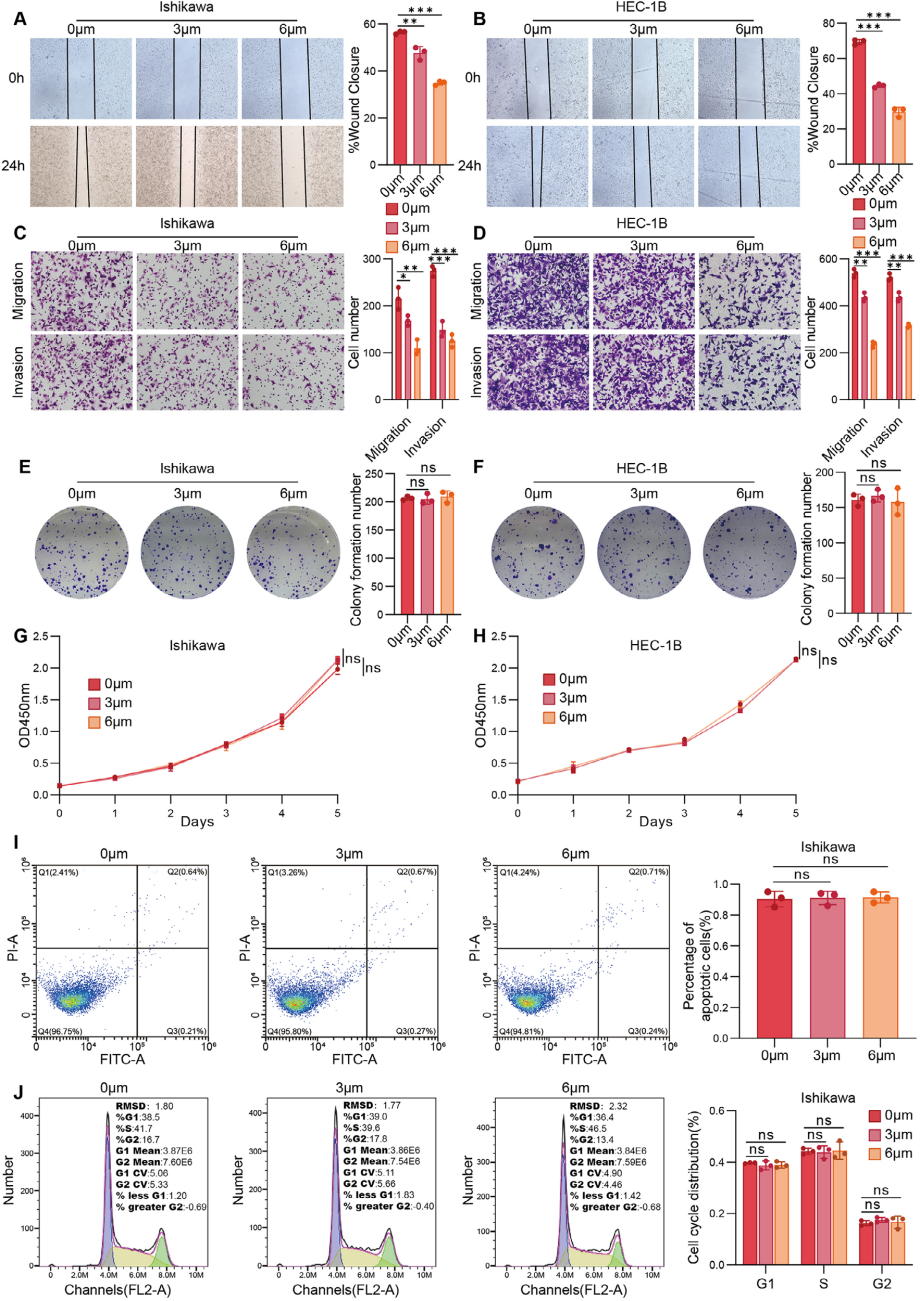
AL398 attenuates metastatic potential of endometrial cancer cells in vitro. (A,B) The migration ability of Ishikawa and HEC‐1B cells was determined by wound‐healing assays after treatment with the drug at concentrations of 0, 3, and 6 µm for 12 h. (C,D) The invasion ability of Ishikawa and HEC‐1B cells was shown by transwell assays. (E,F) The effect of AL398 on the proliferation of Ishikawa and HEC‐1B cells was detected in colony‐formation assays. (G,H) The effect of AL398 on the proliferation of Ishikawa and HEC‐1B cells was detected in CCK‐8 proliferation assay. (I,J) Apoptosis (I) and cell cycle (J) of Ishikawa and HEC‐1B cells were detected by flow cytometry. All quantitative data are presented as mean ± SD from three independent experiments. ^*^ *p* < 0.05, ^**^ *p* < 0.01, ^***^ *p* < 0.001.

To investigate whether AL398 modulates endometrial carcinoma cell proliferation, we performed colony formation and CCK‐8 assays. Results from colony formation assays revealed no significant influence of AL398 on the proliferation of Ishikawa and HEC‐1B cells (Figure 8E,F). Similarly, the CCK‐8 proliferation curves indicated that AL398 treatment did not elicit a statistically significant effect on the proliferation rate relative to the control (Figure 8G,H). Subsequently, the effect of AL398 on apoptosis was evaluated. Annexin V/PI double staining coupled with flow cytometry revealed no significant difference in apoptotic cell numbers after AL398 treatment in Ishikawa cells (Figure 8I). We next examined cell cycle distribution by flow cytometry. Analysis showed no statistically significant alteration in the proportion of S‐phase cells in Ishikawa cells following AL398 treatment at different concentrations compared to the control (Figure 8J). Therefore, these results indicate that AL398 does not significantly affect proliferation, cell cycle progression, or apoptosis in endometrial carcinoma cells. Unlike its negligible effect on the cell cycle, AL398 treatment profoundly influenced EMT markers in Ishikawa cells. Time‐course analysis showed a progressive decrease in mesenchymal markers (ZEB1, N‐cadherin, Vimentin, and Snail) and an increase in E‐cadherin. Furthermore, dose‐dependent experiments confirmed that higher concentrations of AL398 more effectively suppress mesenchymal markers while promoting E‐cadherin expression (Figure 9A). These results demonstrate that AL398 inhibits the metastatic potential of EC cells primarily by modulating the EMT process, rather than affecting cell cycle dynamics. In summary, these results indicate that AL398 functions as a potent inhibitor of endometrial carcinoma metastasis in vitro by effectively suppressing cell migration, invasion, and EMT processes.

**FIGURE 9:**
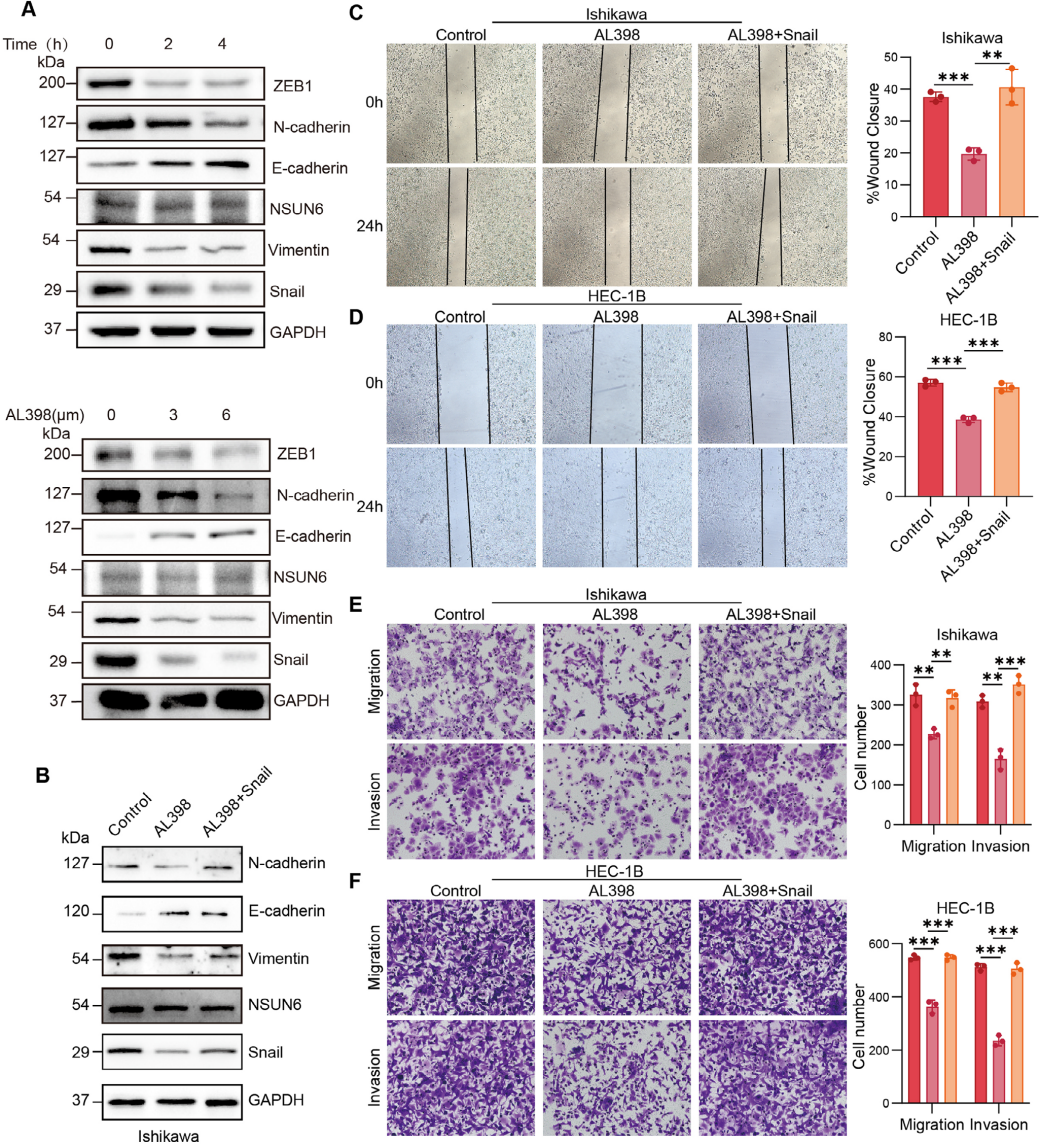
Snail overexpression counteracts the anti‐metastatic effect of the NSUN6 inhibitor. (A) Western blotting was used to detect the effects of AL398 treatment with different durations and concentrations on the expression of epithelial‐mesenchymal transition (EMT)‐related proteins. (B) Expression of EMT marker proteins was analyzed by Western blot in Ishikawa cells under different treatments. (C,D) Cell migration ability was assessed by wound healing assay in Ishikawa (C) and HEC‐1B (D) cells under different treatments. (E,F) Cell migration and invasion abilities were evaluated by Transwell assay in Ishikawa (E) and HEC‐1B (F) cells under different treatments. All quantitative data are presented as mean ± SD from three independent experiments. ^*^ *p* < 0.05, ^**^ *p* < 0.01, ^***^ *p* < 0.001.

### 2.8 Snail Overexpression Counteracts the Anti‐Metastatic Effect of the NSUN6 Inhibitor

To further validate the NSUN6–Snail regulatory axis, we performed rescue experiments in Ishikawa and HEC‐1B cells using a NSUN6‐specific inhibitor alone or in combination with Snail overexpression. Western blot analysis revealed that NSUN6 inhibition markedly reduced Snail expression and reversed the EMT process, characterized by upregulation of epithelial markers and downregulation of mesenchymal markers. Notably, concurrent ectopic expression of Snail partially restored the EMT phenotype that had been attenuated by NSUN6 inhibition, confirming that Snail overexpression compensates for the loss of NSUN6 function (Figure 9B). Functionally, wound healing and Transwell assays demonstrated that NSUN6 inhibitor treatment significantly suppressed the migration and invasion of both cell lines. However, this inhibitory effect was substantially reversed upon Snail co‐overexpression (Figure 9C–F). Together, these rescue experiments indicate that the NSUN6‐specific inhibitor constrains EMT and metastatic potential in endometrial cancer cells through downregulation of Snail, and that exogenous Snail expression effectively counteracts this inhibitory phenotype. These findings further reinforce the central conclusion that NSUN6 promotes endometrial cancer progression via Snail.

### 2.9 In Vivo Safety and Antitumor Efficacy Evaluation of AL398

To evaluate the in vivo toxicity and therapeutic efficacy of AL398, we first determined the maximum tolerated dose and lethal dose (LD50) in mice (Figure 10A). The calculated LD50 of AL398 was 19.25 mg/kg. Survival analysis (Figure 10B) and body weight monitoring (Figure 10C) showed that a dose of 12 mg/kg was well‐tolerated with no significant impact on animal health compared to the control group. Furthermore, biochemical analysis revealed that 12 mg/kg of AL398 did not significantly alter serum levels of BUN, Cre, ALT, or AST, suggesting minimal liver and kidney toxicity (Figure 10D). Gross anatomical observation (Figure 10E) and H&E staining (Figure 10F) of major organs, including the heart, liver, spleen, lung, kidney, and stomach, confirmed the absence of apparent pathological damage. Finally, in the mouse xenograft model, AL398 treatment resulted in a significant, dose‐dependent suppression of tumor growth (Figure 10G). Notably, both the 8 mg/kg and 12 mg/kg doses demonstrated effective inhibition of tumor progression, which was further confirmed by the reduced size and weight of the excised tumor specimens (Figure 10H). These findings indicate that NSUN6 inhibition exerts a potent anti‐tumor effect in vivo, providing a solid foundation for evaluating the therapeutic potential of this pathway in endometrial cancer.

**FIGURE 10:**
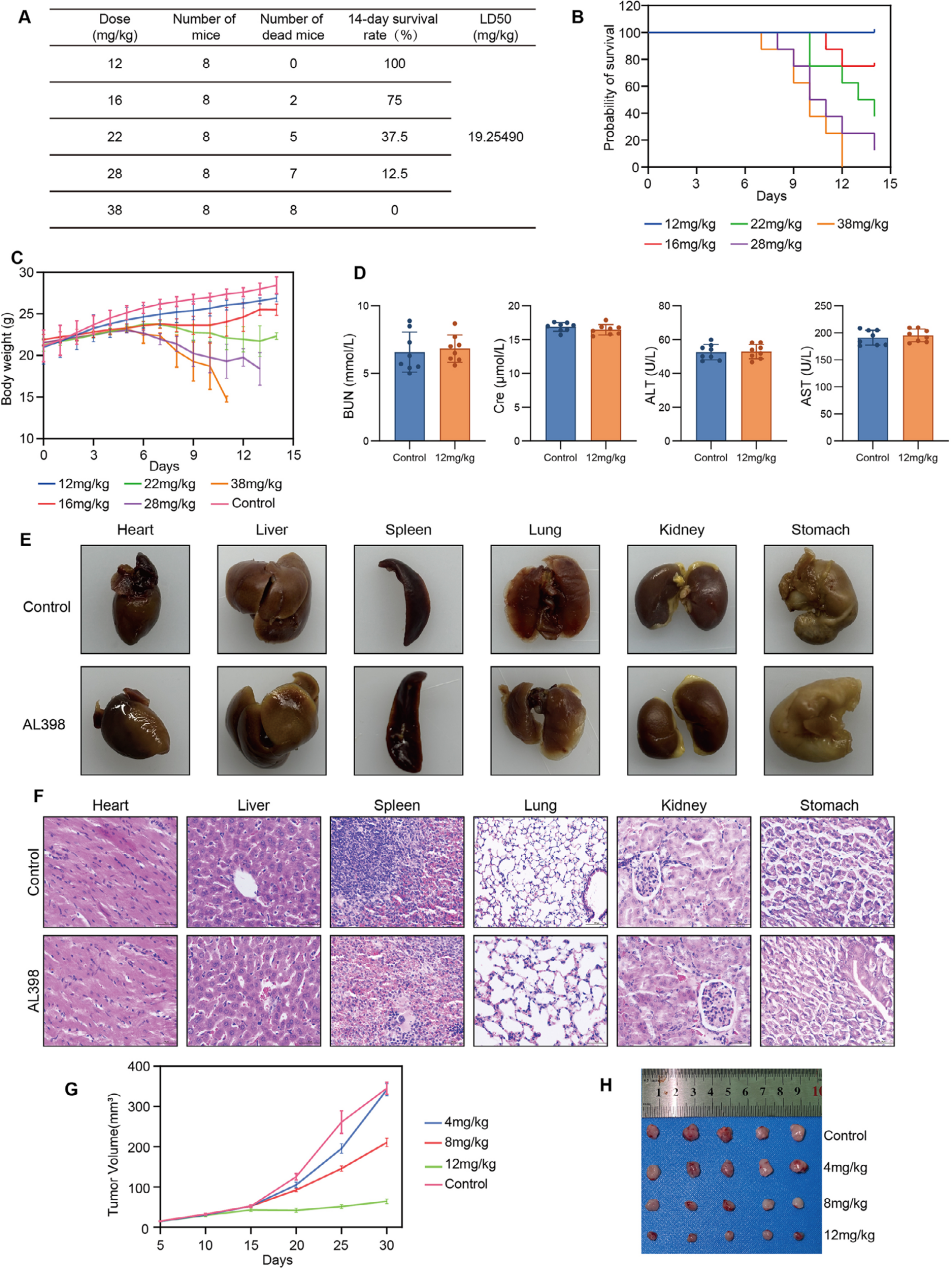
In Vivo Safety and Antitumor Efficacy Evaluation of AL398. (A) Dose grouping, mortality data, and calculated LD50 in mice toxicity tests. (B) Survival curves of mice treated with different drug doses. (C) Body weight changes in mice treated with various drug doses. (D) Liver and kidney function indicators (BUN, Cre, ALT, AST) in mice treated with 12 mg/kg compared to the control group were analyzed using biochemical assays. (E) Representative gross photographs of organs from control and AL398‐treated mice post‐dissection. (F) Representative H&E‐stained sections of major organs from control and AL398‐treated mice. (G) Growth curves of tumor volumes in mice treated with different drug doses. (H) Morphological observation of tumor tissues from each group at the end of the experiment.

### 2.10 AL398 Inhibits Endometrial Cancer Metastasis In Vivo by Targeting the NSUN6/m5C/Snail Pathway

To clarify the in vivo function of the AL398/NSUN6/m5C/Snail axis in the metastasis and invasion of endometrial cancer, we conducted a series of validation experiments using the orthostatic uterine tumor transplantation model and the splenic injection metastasis model (Figure 11I,J). The results of the anterior‐position model showed that, compared with the control group, overexpression of NSUN6 significantly increased tumor volume (Figure 11A and Figure S1G). Histopathological analysis further revealed distinct patterns of cancer cell infiltration between groups. In the control group, cancer cells were observed invading the uterine muscle layer; in contrast, the NSUN6‐overexpression group exhibited infiltration into the skeletal muscle layer. Moreover, while lymph nodes appeared normal in the control group, the NSUN6‐overexpression group showed clear cancer cell invasion into regional lymph nodes (Figure 11B). These findings indicate that overexpression of NSUN6 promotes deeper tissue invasion and facilitates lymphatic metastasis. Subsequently, we evaluated the therapeutic potential of the NSUN6 inhibitor AL398. Bioluminescence imaging revealed that treatment with AL398 markedly suppressed tumor growth and metastatic spread in orthotopic injection model compared to the control group (Figure 11C). Consistent with these functional outcomes, Western blot (Figure 11D) and IHC staining (Figure 11E) analysis further demonstrated that AL398 treatment reverses the NSUN6‐induced epithelial‐mesenchymal transition (EMT) phenotype, characterized by the downregulation of mesenchymal markers (ZEB1, N‐cadherin, Vimentin, and Snail) and the restoration of E‐cadherin expression. Finally, to confirm that this suppression is mediated by the NSUN6/Snail axis, we performed rescue experiments. Bioluminescence imaging in the orthotopic injection model showed that while NSUN6 overexpression enhanced metastasis, this effect was significantly reversed by the knockdown of Snail (Figure 11F). This metastatic suppression was further validated by the reduced tumor burden in the NSUN6/sh‐Snail co‐transfection group compared to the NSUN6‐overexpression group (Figure 11G). Collectively, these data establish that NSUN6 promotes EC metastasis via the Snail‐mediated EMT pathway and that AL398 effectively blocks this process in vivo.

**FIGURE 11:**
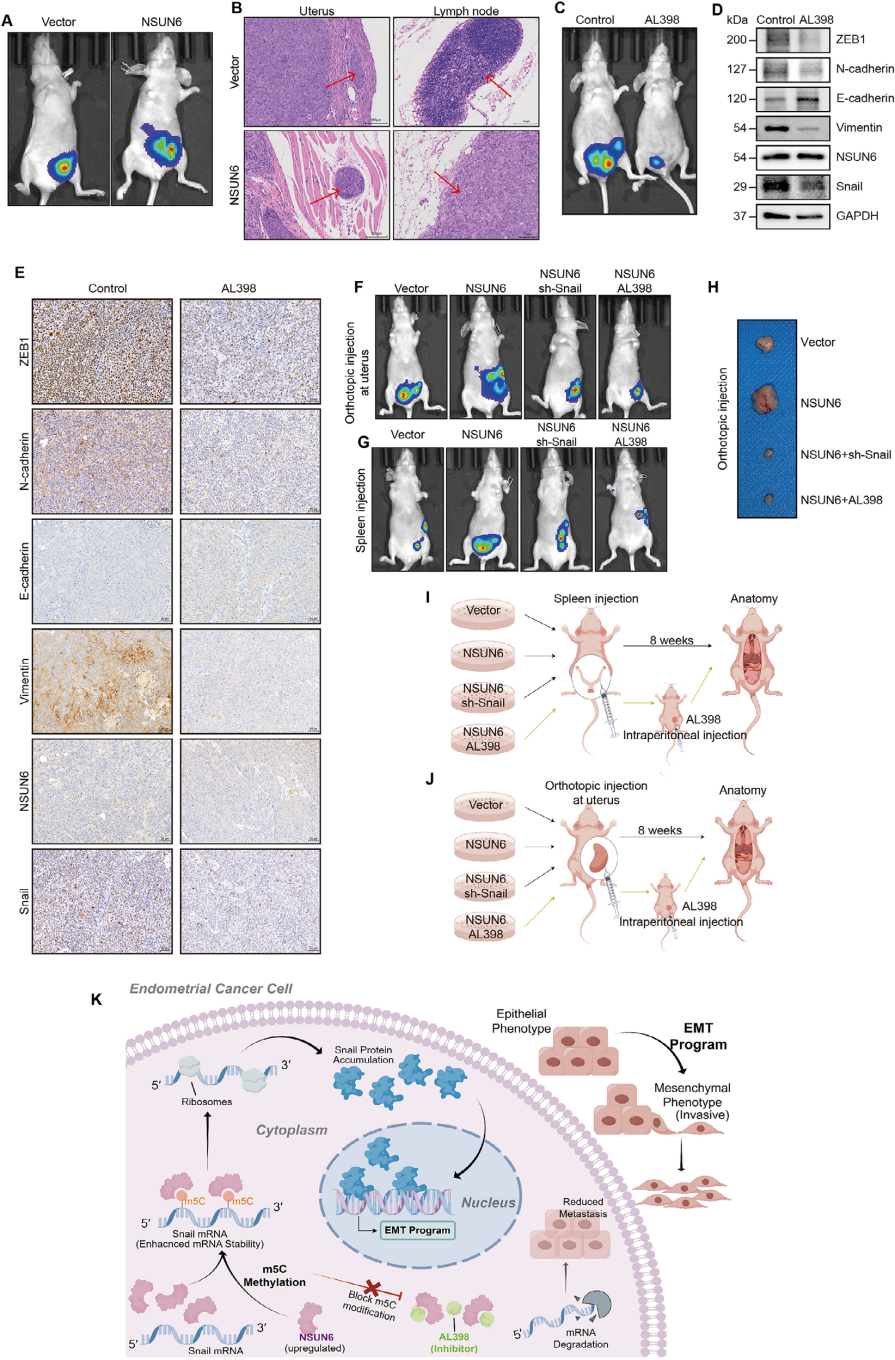
AL398 inhibits endometrial cancer metastasis in vivo by targeting the NSUN6/m5C/Snail pathway. (A) Orthotopic tumor and local invasion schematic. (B) Histology of uterine tissue and regional lymph nodes. (C) The inhibitory effect of AL398 treatment on tumor growth in mice was observed using an in vivo animal imaging system. (D) The effect of AL398 treatment on the expression of EMT‐related proteins in tumor tissues was detected by Western blotting. (E) The expression levels of related proteins in tumor tissues after AL398 treatment were analyzed by immunohistochemistry (IHC) staining. (F,G) In vivo bioluminescence images of mice bearing orthotopic uterine tumors (F) and liver metastases (G). (H) Morphological appearance of tumor tissues from mice in different treatment groups within an orthotopic injection model. (I,J) Schematic diagrams of in vivo models: orthotopic uterine implantation model (I) and spleen injection liver metastasis model (J). (K) Schematic diagram of the AL398/NSUN6/m5C/Snail /EMT axis and its role in regulating the progression of endometrial cancer.

## 3 Discussion

Epigenomic transcriptomics, also known as post‐transcriptional RNA modifications. Recent studies have demonstrated the immense potential of epigenomic transcriptomics in tumor progression research [22, 23, 24]. Although the regulatory networks of RNA modifications have been preliminarily elucidated in multiple tumor progression studies, including brain tumors [25, 26], tumor immune evasion [27, 28, 29], hematologic malignancies [30, 31], their functional roles in EC remain to be thoroughly explored. Endometrial cancer ranks among the most prevalent gynecological malignancies globally, with both incidence and mortality rates showing a persistent upward trend in recent years [32]. Particularly concerning is the limited treatment options and poor prognosis often faced by patients with advanced or recurrent endometrial cancer due to tumor invasion and metastasis [5]. Therefore, in‐depth exploration of the molecular mechanisms underlying endometrial carcinoma invasion and metastasis, coupled with the identification of effective therapeutic targets, has become an urgent need in this field. In this study, we found that the m5C methyltransferase NSUN6 is significantly upregulated in endometrial cancer tissues, and its expression level is closely correlated with the malignant degree of the tumor and poor prognosis, providing new clinical evidence for the role of m5C modification in tumors.

In recent years, targeting RNA modifying enzymes has emerged as a significant research direction in the field of cancer treatment. Taking m6A modification as an example, the inhibitor FB23‐2 of its demethylase FTO has demonstrated remarkable therapeutic potential in acute myeloid leukemia, confirming the feasibility of interfering with tumor progression by regulating RNA methylation levels [33]. Similarly, other studies have also shown that small molecule inhibitors of ALKBH5 can inhibit tumor growth by affecting m6A modification [34, 35, 36], further highlighting the pivotal role of the RNA methylation regulatory network in cancer treatment. It is noteworthy that, despite significant advancements in m6A targeted therapy, research on small molecule inhibitors targeting m5C modifying enzymes remains relatively lagging. Against this backdrop, this study shifts its focus to another important type of RNA modification, m5C. This study identified the NSUN6‐specific inhibitor AL398 through computer‐aided drug design screening. In vivo and in vitro functional experiments simultaneously confirmed that the small molecule inhibitor AL398 effectively inhibits tumor cell invasion and metastasis mediated by this axis. This aligns with recent anti‐tumor strategies targeting RNA modifying enzymes, such as FTO [37, 38], and METTL3 [39, 40], further validating the feasibility of the epigenome as a therapeutic target.

Previous studies have shown that NSUN family proteins can regulate the stability of target mRNA through m5C modification [41]. Based on this, we further reveal that NSUN6 specifically catalyzes the m5C modification of Snail mRNA, thereby enhancing its stability and promoting the EMT process. This discovery not only extends the regulatory mechanism of m5C modification in tumor metastasis but also echoes the known research on NSUN2 regulating oncogene expression through a similar mechanism [42]. Besides, the NSUN6 inhibitor AL398 effectively inhibits the NSUN6/m5C/Snail axis function both in vitro and in vivo, thereby blocking tumor metastasis. This work not only provides a new targeting strategy for the treatment of endometrial cancer but also offers important experimental evidence for expanding RNA modification‐targeted therapy to the m5C field, aligning closely with the current trend of targeted RNA modification therapy. The core breakthrough of this study lies in our revelation of a novel NSUN6/m5C/Snail regulatory axis and the development of targeted therapeutic strategies for it.

From a clinical‐translational perspective, the NSUN6/m5C/Snail axis offers a promising multi‐layered strategy for therapeutic intervention. At the monotherapy level, AL398 could enable precision targeting for advanced patients exhibiting NSUN6 overexpression or dysregulated m5C modification. In combination regimens, this inhibitor may synergize with chemotherapy, radiotherapy, or immune checkpoint inhibitors by reversing the EMT phenotype, thereby overcoming drug resistance and enhancing treatment efficacy. Furthermore, abnormal activation of this pathway could serve as an early biomarker for metastatic risk, allowing preventive interventions in high‐risk patients. These directions together constitute a complete targeted intervention system from early diagnosis to late‐stage treatment, providing new ideas for clinical practice. Beyond the current findings, the potential of patient‐derived organoids (PDOs) and patient‐derived xenografts (PDXs) represents a vital direction for future research. These models, which faithfully retain the heterogeneous genetic and epigenetic landscapes of primary endometrial tumors, offer a robust platform for evaluating the clinical efficacy of AL398. Integrating NSUN6‐targeted therapy into such personalized models could further validate its therapeutic index across diverse molecular subtypes of endometrial cancer, providing a more precise roadmap for translation from bench to bedside.

In summary, our work elucidates that NSUN6 upregulates the stability of Snail mRNA through m5C modification, thereby initiating the EMT program and endowing tumor cells with the ability to migrate and invade. The ultimate realization of this mechanism is the local invasion and highly characteristic lymphatic metastasis of endometrial cancer. More importantly, we have provided a proof‐of‐concept therapeutic tool for this mechanism – a highly effective small molecule inhibitor, AL398. These findings not only deepen our understanding of the biological function of m5C but also lay a solid theoretical and experimental foundation for the development of targeted therapies for advanced or recurrent endometrial cancer.

## 4 Materials and Methods

### 4.1 Cell Culture and Reagents

ESC human normal endometrial cell lines and a panel of human endometrial carcinoma cell lines (HEC‐1B, HEC‐1A, Ishikawa, KLE, RL95‐2) were obtained from Wuhan Pricella Biotechnology Co., Ltd. ESC cells were cultured in human endometrial stromal cell complete medium (Pricella, Wuhan). Ishikawa cells were cultured in DMEM medium (Gibco, USA). KLE cells and RL95‐2 cells were cultured in DMEM/F12 medium, HEC‐1B cells in MEM (containing NEAA) medium, and HEC‐1A cells in McCoy's 5A medium. All tumor cell cultures utilized media supplemented with 10% fetal bovine serum (Clark, USA) and 1% penicillin‐streptomycin (Gibco, USA). Cells were cultured in incubators maintained at 37 °C, 5% CO_2_, and 5% humidity. For in vitro experiments, cells were treated with varying concentrations of MG‐132 (MCE, 13259) and Cycloheximide (MCE, 12320).

### 4.2 Dot Blot

The extracted RNA samples were evaluated for concentration and purity, followed by dilution to an appropriate concentration using RNase‐free PBS. Subsequently, the samples were applied onto nitrocellulose membranes at a volume of 2 µL per spot. Post‐application, the membrane was allowed to air‐dry at room temperature for 10 min. The RNA was then immobilized using a UV crosslinker set at a wavelength of 254 nm and an energy level of 120 mJ/cm^2^. The membrane was subsequently blocked at room temperature for 1 h in PBST containing 1% BSA. It was then incubated overnight at 4°C with an anti‐m5C monoclonal antibody (e.g., Proteintech 68301‐1‐Ig, diluted 1:5000 in 1% BSA/PBST). Following four washes with PBST, the membranes were incubated at room temperature for 1 h with a Goat anti‐Mouse IgG (H+L) Secondary Antibody conjugated to HRP (Abcam, ab228307). After additional washes, the membranes were developed using an ECL substrate. Total RNA staining with methylene blue was employed as the loading control.

### 4.3 Western blot

Cells were lysed using RIPA buffer supplemented with protease inhibitors (China Yamay) and EDTA (Beyotime, China) to facilitate protein extraction. Protein concentration was subsequently determined via the BCA assay. Proteins were resolved on SDS‐PAGE gels and transferred onto PVDF membranes (Millipore, USA). Following blocking, the membranes were incubated overnight at 4 °C with primary antibodies, then incubated for 1 h at room temperature with secondary antibodies, with TBST washes performed between steps. Protein bands were visualized using BeyoECL Star (Beyotime, China) and imaged using the Tanon 4600 system (Tanon, China). The antibodies employed included: Anti‐GAPDH (Abways, AB0037), Anti‐NSUN6 (Proteintech, 17240‐1‐AP), Anti‐SNAI1 (Proteintech, PK30014), Anti‐E‐cadherin (Proteintech, PK30014), Anti‐N‐cadherin (Abways, PK30014), Anti‐Vimentin (Abways, PK30014), Anti‐ZEB1 (Proteintech, 21544‐1‐AP), Anti‐rabbit IgG (Abways, AB0101), and Anti‐mouse IgG (Abways, AB0102).

### 4.4 Transwell and Wound Healing Assays

Researchers used Transwell chambers (Corning, USA) to evaluate cell migration and invasion. For the migration assay, 4 × 10^4^ cells suspended in 200 µL of serum‐free medium were introduced into the upper chamber, while 500 µL of medium supplemented with 20% fetal bovine serum (FBS) was added to the lower chamber to serve as a chemoattractant. Following several hours of incubation, the cells that migrated to the lower surface were fixed with paraformaldehyde, stained with crystal violet, and subsequently imaged. The invasion assay was conducted in a similar manner, with the modification that the upper chamber was pre‐coated with Matrigel (Yeasen, China). Cells were cultured in 6‐well plates for the wound healing assay until they reached roughly 90% confluence. A linear scratch was made using a sterile 10 µL pipette tip, and an image was taken immediately (0 h). Following several hours of incubation in a serum‐free medium, the wound area was re‐imaged to evaluate the migration of cells.

### 4.5 Cell Counting Kit‐8 (CCK‐8)

In 96‐well plates, cells were planted at a density of 2000 per well and maintained for certain time frames. Subsequently, 10 µL of CCK‐8 reagent from Beyotime, China, was combined with 90 µL of complete medium, added to each well, and incubated at 37°C for 2 h. Absorbance was measured at 450 nm using a microplate reader.

### 4.6 Colony Formation

In an atmosphere containing 5% CO_2_, cells were plated in 6‐well plates at a density of 1000 cells per well and incubated at 37°C for 10–14 days. The colonies produced were treated with 4% paraformaldehyde, stained with 0.1% crystal violet, and counted manually.

### 4.7 5‐Ethynyl‐2′‐Deoxyuridine (EdU) Assay

Cell proliferation was evaluated utilizing an EdU assay kit (Beyotime, China) in accordance with the manufacturer's protocol. Specifically, cells were exposed to 10 µm EdU for a duration of 2 h, subsequently fixed with 4% paraformaldehyde, and permeabilized using 0.1% Triton X‐100. Following this, the cells were treated with Click Reaction Solution for 30 min under dark conditions, and the nuclei were counterstained with Hoechst 33342. Fluorescence images were captured using an inverted fluorescence microscope (Olympus, Tokyo, Japan).

### 4.8 Flow cytometry

Subsequent to the respective treatments, cells cultured in 6‐well plates were collected and subjected to dual analyses for apoptosis and cell cycle progression. These assays were conducted utilizing the Annexin V‐FITC Apoptosis Detection Kit and the PI Cell Cycle and Apoptosis Analysis Kit (both procured from Beyotime Biotechnology, China), following the protocols specified by the manufacturer. Flow cytometric analysis was promptly performed using a DxFLEX flow cytometer (Beckman Coulter, USA), and the resulting data were analyzed using FlowJo software.

### 4.9 RT‐qPCR

Total RNA was isolated from the cells utilizing the FastPure Cell/Tissue Total RNA Isolation Kit V2 (Vazyme Biotech Co., Ltd). Following RNA extraction, complementary DNA (cDNA) was synthesized using the Hifair III 1st Strand cDNA Synthesis SuperMix for quantitative PCR (qPCR) (Yeasen, China). Subsequently, quantitative real‐time PCR (qPCR) was conducted employing the Hieff qPCR SYBR Green Master Mix (YEASEN, China) to assess mRNA expression levels. The relative expression of mRNA was quantified using the 2−ΔΔCT method, with GAPDH serving as the internal reference gene.

### 4.10 RNA Immunoprecipitation (RIP) and Methylated RNA Immunoprecipitation (MeRIP)

The interaction between NSUN6 and Snail was examined using an RNA immunoprecipitation kit (Geneseed, Guangzhou, China). Briefly, a total of 2 × 10^7^ cells were lysed with RIP lysis buffer and incubated with magnetic beads conjugated to anti‐IgG (Abcam, ab172730) and anti‐Snail antibodies. The RNA co‐precipitated in this procedure was subsequently analyzed through RT‐qPCR. IgG served as a negative control to rule out non‐specific binding. Furthermore, MeRIP experiments were performed utilizing the m5C MeRIP kit from CloudSeq Biotech Inc. (Shanghai, China), followed by RT‐qPCR analysis of the extracted RNA.

### 4.11 Immunohistochemistry (IHC) Staining

Paraffin‐embedded tissue sections were initially subjected to baking, followed by dewaxing in xylene and rehydration through a graded ethanol series. Antigen retrieval was conducted by heating the slides in a citrate buffer (0.01 m, pH 6.0). To inhibit endogenous peroxidase activity, the sections were incubated with 3% hydrogen peroxide (H_2_O_2_) and subsequently treated with goat serum for 20 min at room temperature. The sections were then incubated with the primary antibody overnight at 4°C. Following three washes with phosphate‐buffered saline (PBS), an enzyme‐labeled secondary antibody (PV‐6000 goat anti‐rabbit IgG/HRP polymer) was applied and incubated for 1 h at room temperature. Staining was developed using a diaminobenzidine (DAB) kit in accordance with the manufacturer's instructions. Hematoxylin was employed for nuclear counterstaining, after which the sections were dehydrated through a graded ethanol series, cleared in xylene, and mounted with neutral gum. For quantitative analysis, the IHC staining was evaluated using a semi‐quantitative scoring system based on both the intensity of staining and the percentage of positive cells. The staining intensity was graded as follows: 0 (negative), 1 (weak), 2 (moderate), and 3 (strong). The percentage of positive cells was scored as: 0 (0%), 1 (1–25%), 2 (26–50%), 3 (51–75%), and 4 (76–100%). The final immunoreactive score (IRS) was calculated by multiplying the intensity score by the percentage score. All immunohistochemical results were independently evaluated and scored by two experienced pathologists who were strictly blinded to the clinical data.

### 4.12 Animals and In Vivo Experiments

All animal experiments were conducted in accordance with the protocols approved by the Institutional Animal Care and Use Committee of Henan Zhongmu Biotech Research Institute (Approval No: ZM(lun)202606018). BALB/c nude mice (6–8 weeks old) were housed in a specific pathogen‐free (SPF) facility under controlled temperature (22 ± 2°C), humidity (50 ± 10%), and a 12‐h light/dark cycle, with ad libitum access to food and water. Sample sizes were determined based on preliminary experiments to ensure statistical power while adhering to the 3R principles.

### 4.13 Median Lethal Dose (LD50) Determination

To establish a safe dosing range, 32 mice were randomly assigned to four groups (*n* = 8/group, equal sex ratio). Mice were administered different doses of the inhibitor (12, 16, 22, 28, and 38 mg/kg) via intraperitoneal injection. Mortality and body weight were monitored daily for 16 days.

### 4.14 Tumor Growth Inhibition Assay

A subcutaneous xenograft model was established by injecting Ishikawa cells (5 × 10^6^) into the flank of female BALB/c nude mice. On day 15 post‐inoculation, mice were randomized into four groups (*n* = 5/group) and treated i.p. with vehicle control or the inhibitor (4, 8, and 12 mg/kg) daily for 15 days. Tumor volumes were calculated as (length × width^2^)/2.

### 4.15 Preliminary In Vivo Bioluminescence Imaging

To observe the effect of the inhibitor on tumor distribution, 6 female BALB/c nude mice were divided into two groups. Mice were inoculated with Luciferase‐labeled Ishikawa cells. The treatment group received 8 mg/kg of the inhibitor, while the control group received an equal volume of vehicle via injection.

### 4.16 Orthotopic and Intrasplenic Metastasis Models

For the orthotopic model, female BALB/c nude mice (6 weeks old) were anesthetized with Avertin (0.1 mL/10 g body weight). A small abdominal incision was made to expose the uterus, and Luciferase‐labeled Ishikawa cells (2 × 10^6^ cells in 100 µL PBS) were injected directly into the uterine horn. For the intrasplenic metastasis model, mice were anesthetized and prepared similarly; an incision was made in the left abdomen to expose the spleen. Luciferase‐labeled Ishikawa cells (1 × 10^6^ cells in 50 µL PBS) were injected into the splenic capsule. To prevent leakage and intraperitoneal dissemination, the injection site was compressed for 30 s. Incisions were closed using surgical sutures. Mice were kept on a warming plate until fully recovered. Tumor dissemination and colonization were monitored via in vivo bioluminescence imaging. Upon study completion, mice were euthanized via cervical dislocation, and tissues were harvested for analysis.

### 4.17 Ethical Oversight and Humane Endpoints

Mice were monitored daily. Humane endpoints were implemented if mice exhibited: (1) lethargy or unresponsiveness; (2) severe respiratory distress; (3) persistent diarrhea or incontinence; (4) inability to access food/water; (5) signs of severe anxiety or distress. Animals reaching these criteria were euthanized via cervical dislocation.

### 4.18 Tissue Collection

Upon the completion of the experiments, animals were euthanized via cervical dislocation. Tumor tissues and major organs were immediately excised, washed with ice‐cold phosphate‐buffered saline (PBS), and measured for size and weight. A portion of the tissue was fixed in 4% paraformaldehyde for histological analysis, while the remaining tissue samples were snap‐frozen in liquid nitrogen and stored at −80 °C for subsequent molecular biology assays, including Western blot and qRT‐PCR

### 4.19 Statistical Analysis

In this study, each experiment was performed a minimum of three times. The resulting experimental data were subjected to statistical analysis and are presented as the mean ±standard deviation. A P‐value of less than 0.05 was considered indicative of statistical significance. Significance levels are represented as follows: “ns” indicates no significance, “*” denotes *p* < 0.05, “**” signifies *p* < 0.01, and “***” represents *p* < 0.001.

## Author Contributions

H.L., J.W. and Q.K. designed this study. X.Z., L.Y., H.C., C.G., H.C., H.L., Y.Z., X.W., Y.L. and R.Z. performed the experiments. X.Z., C.G., Z.H. and Y.C. performed the data analysis. X.Z., Y.H., and J.W. reviewed the pathological data. X.Z. drafted the manuscript. H.L., J.W. and Q.K. reviewed the manuscript. All authors reviewed and approved the final manuscript.

## Fundings

This research received financial support from the Henan Clinical Medical Scientist Program (Grant HNCMS202514). Additional funding was provided by the National Natural Science Foundation of China (Grants 82607625, 82172351, 82173018, 82370113 and 31900895), the Natural Science Foundation of Henan Province (Grant 232300421054, 262300421153), the Henan Province Science and Technology Research Project (Grant 262102310052), the Medical Science and Technology Provincial and Ministerial Co‐construction Project of Henan Province (Grant SBGJ202403034), as well as the Zhengzhou University Graduate Education Research Project (Grants YJSJY2025182 and YJSJY2025165). This study was also supported by Joint Fund of Henan Province Science and Technology R&D Program (Grant 235200810085 and 235200810039).

## Ethics Statement

This study was conducted in accordance with the ethical standards of the Declaration of Helsinki and was approved by the Ethics Committee of the Third Affiliated Hospital of Zhengzhou University.

## Conflicts of Interest

The authors declare no conflicts of interest.

## Supporting information




**Supporting File**: advs77359‐sup‐0001‐SuppMat.docx.

## Data Availability

The data that support the findings of this study are available in the supplementary material of this article.

## References

[1] P. Morice , A. Leary , C. Creutzberg , N. Abu‐Rustum , and E. Darai , “Endometrial Cancer,” Lancet 387 (2016): 1094–1108, 10.1016/S0140-6736(15)00130-0. 26354523

[2] M. Sbarra , M. Lupinelli , B. OR , A. M. Venkatesan , and S. Nougaret , “Imaging of Endometrial Cancer,” Radiologic Clinics of North America 61 (2023): 609–625, 10.1016/j.rcl.2023.02.007. 37169427

[3] R. L. Siegel , T. B. Kratzer , A. N. Giaquinto , H. Sung , and A. Jemal , “Cancer Statistics,” CA: A Cancer Journal for Clinicians 75 (2025): 10. 10.3322/caac.21871 PMC11745215 39817679

[4] J. McEachron , L. Marshall , N. Zhou , et al., “Evaluation of Survival, Recurrence Patterns and Adjuvant Therapy in Surgically Staged High‐Grade Endometrial Cancer with Retroperitoneal Metastases,” Cancers 13 (2021): 2052, 10.3390/cancers13092052. PMC8123054 33922792

[5] S. Acharya , M. L. Hensley , A. C. Montag , and G. F. Fleming , “Rare Uterine Cancers,” The Lancet Oncology 6 (2005): 961–971, 10.1016/S1470-2045(05)70463-0. 16321764

[6] F. Amant , M. R. Mirza , M. Koskas , and C. L. Creutzberg , “Cancer of the Corpus Uteri,” International Journal of Gynaecology and Obstetrics 131 (2015): S96–S104. 10.1016/j.ijgo.2015.06.005 26433681

[7] F. Tronconi , C. Nero , E. Giudice , et al., “Advanced and Recurrent Endometrial Cancer: State of the Art and Future Perspectives,” Critical Reviews in Oncology/Hematology 180 (2022): 103851, 10.1016/j.critrevonc.2022.103851. 36257537

[8] R. Su , L. Dong , Y. Li , et al., “Effective Novel Fto Inhibitors Show Potent Anti‐Cancer Efficacy and Suppress Drug Resistance,” Blood 134 (2019): 233, 10.1182/blood-2019-124535.

[9] R. Su , L. Dong , Y. Li , et al., “Targeting FTO Suppresses Cancer Stem Cell Maintenance and Immune Evasion,” Cancer Cell 38 (2020): 79–96.e11, 10.1016/j.ccell.2020.04.017. PMC7363590 32531268

[10] T. Jiang , Y. Xiao , J. Zhou , et al., “Arbutin Alleviates Fatty Liver by Inhibiting Ferroptosis via FTO/SLC7A11 Pathway,” Redox Biology 68 (2023): 102963, 10.1016/j.redox.2023.102963. PMC10694775 37984229

[11] E. Yankova , W. Blackaby , M. Albertella , et al., “Small‐Molecule Inhibition of METTL3 as a Strategy Against Myeloid Leukaemia,” Nature 593 (2021): 597–601, 10.1038/s41586-021-03536-w. PMC7613134 33902106

[12] X. Zhou , X. Yang , S. Huang , et al., “Inhibition of METTL3 Alleviates NLRP3 Inflammasome Activation via Increasing Ubiquitination of NEK7,” Advanced Science 11 (2024): 2308786, 10.1002/advs.202308786. PMC11234428 38696610

[13] H. Yin , Z. Ju , X. Zhang , et al., “Inhibition of METTL3 in Macrophages Provides Protection Against Intestinal Inflammation,” Cellular & Molecular Immunology 21 (2024): 589–603, 10.1038/s41423-024-01156-8. PMC11143309 38649449

[14] R. J. Liu , T. Long , J. Li , H. Li , and E. D. Wang , “Structural Basis for Substrate Binding And Catalytic Mechanism of a Human RNA:M5C Methyltransferase NSun6,” Nucleic Acids Research 45 (2017): 6684–6697, 10.1093/nar/gkx473. PMC5499824 28531330

[15] M. Yu , M. Ni , F. Xu , et al., “NSUN6‐Mediated 5‐Methylcytosine Modification of NDRG1 mRNA Promotes Radioresistance in Cervical Cancer,” Molecular Cancer 23 (2024): 139, 10.1186/s12943-024-02055-2. PMC11225205 38970106

[16] J. Guo , B. Wu , S. Wang , D. Huang , and Y. Hu , “NSUN6 Promotes Gastric Cancer Progression by Stabilizing CEBPZ mRNA in a m5C‐Dependent Manner,” Applied Biochemistry and Biotechnology 197 (2025): 7296–7313, 10.1007/s12010-025-05367-1. 40928700

[17] Y. Cui , P. Lv , and C. Zhang , “NSUN6 Mediates 5‐Methylcytosine Modification of METTL3 and Promotes Colon Adenocarcinoma Progression,” Journal of Biochemical and Molecular Toxicology 38 (2024): 23749, 10.1002/jbt.23749. 38800929

[18] X. Tong , S. Wang , Z. Lei , et al., “MYOCD and SMAD3/SMAD4 Form a Positive Feedback Loop and Drive TGF‐β‐Induced Epithelial–Mesenchymal Transition in Non‐Small Cell Lung Cancer,” Oncogene 39 (2020): 2890–2904, 10.1038/s41388-020-1189-4. 32029901

[19] R. Song , H. Song , Y. Liang , et al., “Reciprocal Activation Between ATPase Inhibitory Factor 1 and NF‐κB Drives Hepatocellular Carcinoma Angiogenesis and Metastasis,” Hepatology 60 (2014): 1659–1673, 10.1002/hep.27312. 25042864

[20] P. Ouyang , K. Li , W. Xu , et al., “METTL3 recruiting M2‐type Immunosuppressed Macrophages by Targeting m6A‐SNAIL‐CXCL2 Axis to Promote Colorectal Cancer Pulmonary Metastasis,” Journal of Experimental & Clinical Cancer Research 43 (2024): 111, 10.1186/s13046-024-03035-6. PMC11007974 38605400

[21] J. Y. Kim , Y. M. Kim , C. H. Yang , S. K. Cho , J. W. Lee , and M. Cho , “Functional Regulation of Slug / Snail2 is Dependent on GSK‐3β‐Mediated Phosphorylation,” The FEBS Journal 279 (2012): 2929–2939, 10.1111/j.1742-4658.2012.08674.x. 22727060

[22] R. Xie , L. Cheng , M. Huang , et al., “NAT10 Drives Cisplatin Chemoresistance by Enhancing ac4C‐Associated DNA Repair in Bladder Cancer,” Cancer Research 83 (2023): 1666–1683, 10.1158/0008-5472.CAN-22-2233. 36939377

[23] V. Ortiz‐Barahona , M. Soler , V. Davalos , et al., “Epigenetic Inactivation of the 5‐Methylcytosine RNA Methyltransferase NSUN7 is Associated With Clinical Outcome and Therapeutic Vulnerability in Liver Cancer,” Molecular Cancer 22 (2023): 83, 10.1186/s12943-023-01785-z. PMC10176850 37173708

[24] M. Janin , V. Davalos , and M. Esteller , “Cancer Metastasis Under the Magnifying Glass of Epigenetics and Epitranscriptomics,” Cancer and Metastasis Reviews 42 (2023): 1071–1112, 10.1007/s10555-023-10120-3. PMC10713773 37369946

[25] A. Z. Huang , A. Delaidelli , and P. H. Sorensen , “RNA Modifications in Brain Tumorigenesis,” Acta Neuropathologica Communications 8 (2020): 64, 10.1186/s40478-020-00941-6. PMC7204278 32375856

[26] L. Kukreja , C. J. Li , S. Ezhilan , V. R. Iyer , and J. S. Kuo , “Emerging Epigenetic Therapies for Brain Tumors,” NeuroMolecular Medicine 24 (2022): 41–49, 10.1007/s12017-021-08691-x. 34677796

[27] W. Li , Y. Hao , X. Zhang , S. Xu , and D. Pang , “Targeting RNA N6‐Methyladenosine Modification: A Precise Weapon in Overcoming Tumor Immune Escape,” Molecular Cancer 21 (2022): 176, 10.1186/s12943-022-01652-3. PMC9454167 36071523

[28] Y. Kong , J. Yu , S. Ge , and X. Fan , “Novel Insight Into RNA Modifications in Tumor Immunity: Promising Targets to Prevent Tumor Immune Escape,” Innovation (Camb) 4 (2023): 100452. 10.1016/j.xinn.2023.100452 PMC10362524 37485079

[29] Z. Zhang , F. Liu , W. Chen , et al., “The importance of N6‐Methyladenosine Modification in Tumor Immunity and Immunotherapy,” Experimental Hematology & Oncology 11 (2022): 30, 10.1186/s40164-022-00281-2. PMC9118853 35590394

[30] L. Yao , H. Yin , M. Hong , et al., “RNA methylation in Hematological Malignancies and its Interactions with Other Epigenetic Modifications,” Leukemia 35 (2021): 1243–1257, 10.1038/s41375-021-01225-1. PMC8102199 33767371

[31] S. Yang , L. Xu , H. Zhuang , F. Li , and Y. Lu , “A New Perspective on Hematological Malignancies: M6A Modification in Immune Microenvironment,” Frontiers in Immunology 15 (2024): 1374390, 10.3389/fimmu.2024.1374390. PMC11168112 38868768

[32] E. J. Crosbie , S. J. Kitson , J. N. McAlpine , A. Mukhopadhyay , M. E. Powell , and N. Singh , “Endometrial Cancer,” The Lancet 399 (2022): 1412–1428, 10.1016/S0140-6736(22)00323-3. 35397864

[33] Y. Huang , R. Su , Y. Sheng , et al., “Small‐Molecule Targeting of Oncogenic FTO Demethylase in Acute Myeloid Leukemia,” Cancer Cell 35 (2019): 677–691.e10, 10.1016/j.ccell.2019.03.006. PMC6812656 30991027

[34] G.‐Q. Lai , Y. Li , H. Zhu , et al., “A Covalent Compound Selectively Inhibits RNA Demethylase ALKBH5 Rather Than FTO,” RSC Chemical Biology 5 (2024): 335–343, 10.1039/D3CB00230F. PMC10989504 38576724

[35] L. Liang , W. Fei , Y. Wang , Z. Zhang , Q. You , and X. Guo , “Discovery of Maleimide Derivatives as m6A Demethylase ALKBH5 Inhibitors,” Bioorganic & Medicinal Chemistry 120 (2025): 118083, 10.1016/j.bmc.2025.118083. 39904196

[36] W.‐L. Fei , Y.‐Z. Wang , Q.‐L. Feng , et al., “Discovery of the Salicylaldehyde‐Based Compound DDO‐02267 as a Lysine‐Targeting Covalent Inhibitor of ALKBH5,” European Journal of Medicinal Chemistry 284 (2025): 117183, 10.1016/j.ejmech.2024.117183. 39740322

[37] Z. Liu , Z. Duan , D. Zhang , et al., “Structure–Activity Relationships and Antileukemia Effects of the Tricyclic Benzoic Acid FTO Inhibitors,” Journal of Medicinal Chemistry 65 (2022): 10638–10654, 10.1021/acs.jmedchem.2c00848. 35793358

[38] H. Huang , X. Li , J. Luo , et al., “FTO Regulates ELK3‐Mediated Metabolic Rewiring and Represents a Unique Therapeutic Target in T cell Leukemia,” Science Advances 11 (2025): adq3052, 10.1126/sciadv.adq3052. PMC12118595 40435251

[39] X. Chen , M. Wang , H. Wang , et al., “METTL3 Inhibitor Suppresses the Progression of Prostate Cancer via IGFBP3/AKT Pathway and Synergizes with PARP Inhibitor,” Biomedicine & Pharmacotherapy 179 (2024): 117366, 10.1016/j.biopha.2024.117366. 39232384

[40] X. Zhou , K. Chai , H. Zhu , et al., “The role of the Methyltransferase METTL3 in Prostate Cancer: A Potential Therapeutic Target,” BMC Cancer 24 (2024): 8, 10.1186/s12885-023-11741-1. PMC10762986 38166703

[41] R. Wang , L. Ding , Y. Lin , et al., “The Quiet Giant: Identification, Effectors, Molecular Mechanism, Physiological and Pathological Function in mRNA 5‐methylcytosine Modification,” International Journal of Biological Sciences 20 (2024): 6241–6254, 10.7150/ijbs.101337. PMC11628344 39664561

[42] S. Liu , B. Xu , and J. Zhao , “NSUN2‐Mediated m5C Modification of PGK1 mRNA Promotes Cell Growth, Invasion, Stemness and Glycolysis in Gastric Cancer,” Cell Cycle 24 (2025): 283–295, 10.1080/15384101.2025.2544829. PMC12439567 40792659

