# Supplementary material for: AL398, A Novel NSUN6 Inhibitor, Attenuates Epithelial‐Mesenchymal Transition and Metastasis in Endometrial Cancer by Abrogating m5C‐Dependent Stabilization of Snail mRNA
Source: Adv Sci (Weinh). 2026 Aug 28:e77359. Online ahead of print. doi: 10.1002/advs.77359 (PMC13525354; doi:10.1002/advs.77359)
Supplement: Supplementary file 1 [file ADVS-9999-e77359-s001.docx]

**Figure S1. NSUN6-m5C-YBX1 axis regulates Snail mRNA stability and malignant progression of endometrial cancer**

**A** Western blotting was used to verify the knockdown efficiency of NSUN6. **B** MeRIP assay was used to examine the effects of NSUN6 expression and AL398 treatment on SNAIL m5C modification and protein binding. **C** The binding abilities of YBX1 and ALYREF to SNAIL were detected by RIP-qPCR. **D** Actinomycin D (ActD) treatment and qPCR were used to analyze the regulation of YBX1 on SNAIL mRNA stability. **E** Western blotting was performed to analyze the effect of NSUN6 knockdown on YBX1 and Snail protein expression. **F** Migration and invasion assays were used to analyze the functional rescue of NSUN6 knockdown by YBX1 overexpression on cellular malignant phenotypes. G Tumor progression was monitored by measuring total photon flux every 5 days over a 35-day period in Vector and NSUN6-overexpressing groups.

**Figure S2. Molecular docking analysis of potential NSUN6 inhibitors**

**A** 3D molecular docking diagram of AG690. **B** 3D molecular docking diagram of AP770. **C** 3D molecular docking diagram of AK968. **D** 3D molecular docking diagram of AL398. **E** 3D molecular docking diagram of AP124. **F** 3D molecular docking diagram of AO476. **G** Representative m5C dot blot images and the corresponding dose-response curve showing the inhibition rate of AL398 at various concentrations. **H** Cellular thermal shift assay (CETSA) was used to evaluate the effect of AL398 on the thermal stability of NOP2, NSUN2, and METTL3. **I** Relative expression levels of Snail were quantified at various time points (0, 0.5, 1, 2, 4, and 8 h) following AL398 treatment. Error bars represent standard deviation.
